# All shallow coastal habitats matter as nurseries for Mediterranean juvenile fish

**DOI:** 10.1038/s41598-021-93557-2

**Published:** 2021-07-16

**Authors:** Adrien Cheminée, Laurence Le Direach, Elodie Rouanet, Patrick Astruch, Adrien Goujard, Aurélie Blanfuné, Denis Bonhomme, Laureline Chassaing, Jean-Yves Jouvenel, Sandrine Ruitton, Thierry Thibaut, Mireille Harmelin-Vivien

**Affiliations:** 1 Faculté des Sciences, Aix Marseille Université, 163 Avenue de Luminy, Case 901, 13288 Marseille, France; 2 Septentrion Environnement, 89 Traverse Parangon, 13008 Marseille, France; 3 GIS Posidonie, OSU Institut Pytheas, Oceanomed, Case 901, Campus de Luminy, 13288 Marseille Cedex 9, France; 4 Aix Marseille Univ., Université de Toulon, CNRS, IRD, MIO UM 110, 13288 Marseille, France; 5 Marseille, France; 6 P2A Développement, 87 Av. F. de Lesseps, Impasse Algrin, 34110 Frontignan, France

**Keywords:** Community ecology, Conservation biology, Ecosystem ecology

## Abstract

Coastal zones are ecosystems of high economic value but exposed to numerous disturbances, while they represent nurseries for many fish species, raising the issue of the preservation of their functions and services. In this context, the juvenile fish assemblages of all types of habitats present in shallow coastal zones were studied on the south-east coast of France using underwater visual censuses in warm (June–July 2014) and cold (April 2015) periods. A total of fourteen habitat types were characterized, which could be grouped into three broad categories, rocky substrates (natural and artificial), sedimentary bottoms with all levels of granulometry, and seagrass beds including *Cymodocea nodosa* and *Posidonia oceanica* meadows; the ecotones or interfaces between the three broad habitat categories were individualized as particular habitat types. The abiotic and biotic descriptors of the 14 habitat types individualized did not vary with time, except for a higher cover percentage and canopy height of macrophytes in the warm period, which increased the three-dimensional structure of some habitats. The taxonomic composition and density of juvenile fish assemblages were analyzed using both multivariate and univariate descriptors, after grouping the 57 fish species recorded into 41 well-individualized taxa. Juvenile fishes were recorded in all habitat types, with higher mean species richness and abundance during the warm than the cold period. The richest habitats in terms of both fish species richness and abundance were the natural rocky substrates and the interfaces between *Posidonia* beds and the other habitats. Although juvenile fish assemblage composition differed among habitat types and between periods, the most abundant fish species were *Atherina* sp., *Sarpa salpa*, Gobiidae, *Symphodus* spp., *Pagellus* spp. and several *Diplodus* species, which colonized 7 up to 14 different habitat types (depending on taxa) during their juvenile life. Most species settled in one or a few specific habitats but rapidly colonized adjacent habitats when growing. This study provided evidence of the role of all types of shallow coastal habitats as fish nurseries and their varying pattern of occupation in space and time by the different juvenile stages. It highlighted the importance of the mosaic of habitats and interfaces for the complete development of all juvenile life stages of fishes, and for the preservation of a high diversity of coastal fish assemblages and fisheries resources in the Mediterranean Sea.

## Introduction

Coastal areas have long been known as high commercial value areas^1, 2^ but also as the zones most impacted by anthropogenic disturbances^3, 4^, including habitat destruction^5^, chemical pollution^6, 7^, artisanal and recreational fishing^8, 9^, and more recently anthropogenic noise pollution^10^. However, coastal zones also represent nursery sites for numerous fishes, including commercial species^11–13^.

Most benthic and demersal fish species present a bipartite life cycle with a dispersive pelagic larval phase and a more sedentary benthic adult phase^14^. Depending on the species-specific planktonic larval phase duration (PLD)^15^, reproduction products may be dispersed on a more or less extensive stretch of coastline, ranging from a few hundred meters to hundreds of kilometers^16–18^. When competent, the surviving larvae metamorphose into juveniles and settle in specific habitats (settlement phase) where they grow for a few months, before being recruited into adult populations (recruitment phase) generally in deeper and more diverse habitats, as juveniles and adults often occupy different habitat types^11, 19, 20^. As the replenishment of local adult fish populations depends on the success of their larval and juvenile phases, juvenile survival in nursery habitats means that they are of paramount importance with regard to the fish life cycle, stock conservation and fisheries exploitation^21–24^. Settlement in nurseries may occur at different times of the year according to species spawning period^25, 26^. Moreover, although some alternatives may exist, the settlement-recruitment process usually follows similar patterns: during settlement, one main or several cohorts of settlers may occur, resulting in a uni- or pluri-modal settlement peak^27, 28^. This peak may be quantified as the density of new settlers per unit area of habitat and is referred to as “settlement intensity” or “settlement success”^20, 29^. The settlement peak is then followed by a period where juveniles grow inside nursery habitats, and during which they may display ontogenic habitat changes, switching between various nursery habitats as they grow and require new resources^19, 20^. Ultimately, surviving juveniles (recruits) may join adult populations and habitats (i.e. recruitment). The quantity of surviving juveniles inside nurseries after an arbitrary period of time following settlement has been used as a measure of “recruitment level”^29^. Beck and collaborators^11, 30^ describe the “nursery value” of a given habitat as a more comprehensive view of these descriptors: the nursery value of a given habitat is the quantity of new individuals produced per unit area and provided to adult populations as an outcome of the combination of four components: the initial number of settlers provided to a nursery, their growth and survival, and their capacity to join the adult population (i.e. functional and structural connectivities) (but see other works for alternative points of views^13, 24^).

Numerous studies have been undertaken on the role of particular shallow coastal habitats as fish nursery sites in the Mediterranean Sea. A few highlighted the effects of environmental characteristics and seasonal variations on juvenile fish assemblages. They notably showed both temporal and spatial partitioning of resources as juvenile settlement of some species occurs in different habitats, and when some species occupy the same juvenile habitat they do it at various time of the year^25, 26, 29, 31–34^. One approach was to focus on one type of habitat, such as coastal lagoons^35, 36^, soft bottoms^37, 38^, *Cymodocea nodosa* meadows^39–42^, *Posidonia oceanica* beds^43–47^, shallow rocky reefs more or less colonized by macrophytes assemblages^28, 31, 32, 48–55^ and shallow heterogeneous rocky substrates^56, 57^. Another approach was to focus on specific fish species such as the gilthead seabream *Sparus aurata*^58^, sparids of the genus *Diplodus*^25, 59–64^, flatfishes such as *Solea solea*^65, 66^, the dusky grouper^67–71^, labrid species^72–75^ or blennies^76^.

While a few studies were focused on multiple habitats^26, 33, 77, 78^, no study has been yet systematically carried out on all habitat types encountered along the coast without any a priori assumption regarding their potential role as nurseries for fishes. The present study was thus designed to explore the potential contribution of all shallow (< 6 m) coastal habitats and their interfaces (i.e. ecotones) as fish nurseries on the coasts of Provence (France, NW Mediterranean), whatever the type and intensity of human pressures. The aims of the present study were to (1) define the environmental characteristics of the habitat types present in shallow coastal habitats and their main temporal variations, (2) characterize the juvenile fish assemblages associated with the different habitats and relate or not their seasonal variations to environmental changes in habitat structure, and (3) determine the potential of the different habitat types as nurseries for juvenile Mediterranean coastal fishes in the frame of coastal protection improvement.

## Material and methods

### Ethics statement

The observational protocol was submitted to the regional authority 'Direction interrégionnale de la mer Méditerranée' (the French administration in charge of Maritime Affairs), which did not require a special permit since no extractive sampling or animal manipulations were performed (only visual censuses in natural habitats), since the study did not involve endangered or protected species and since the surveyed locations were not privately owned.

### Sites and sampling methods

**Figure 1:**
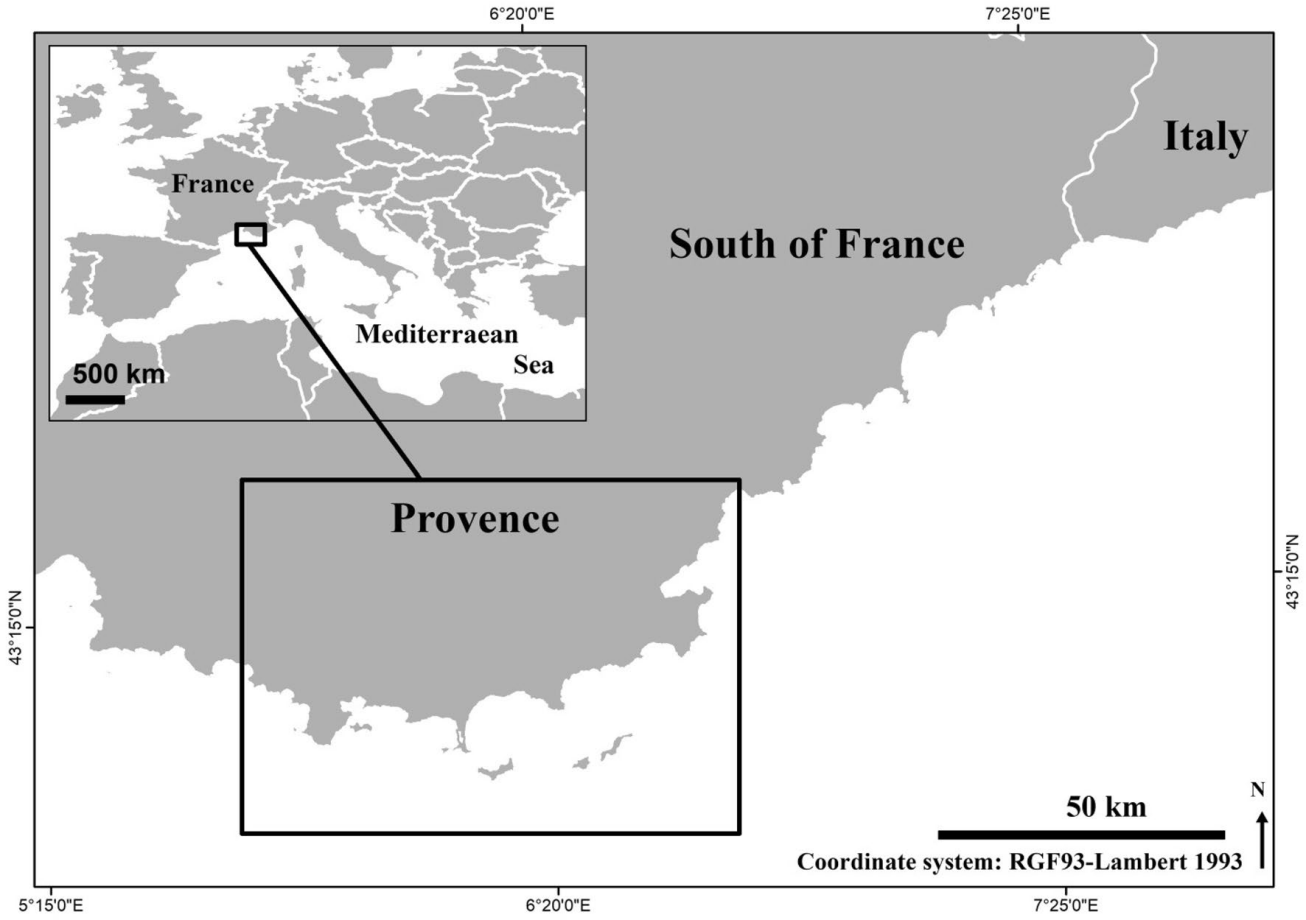
Map of the studied area: 42 stations were sampled along the 100 km stretch of the studied portion of the French Provence coastline (black rectangle). The map was drawn using free and open source software Inkscape 0.91 (https://inkscape.org/en/) and QGIS 2.14 (http://www.qgis.org/). Map was drawn by authors using online Standard tile layer from OpenStreetMap data as background model (© OpenStreetMap contributors), available under ODbL licence (CC-BY-SA) at http://www.openstreetmap.org/.

Juvenile fish were monitored by underwater visual censuses (UVC)^79^ in a wide variety of habitats from 0.5 to 6 m depth at stations randomly spread along a 100 km long stretch of the Provence coastline (Fig. 1). A random sampling design was adopted to encompass the natural characteristics and spatial variability of shallow coastal habitats during the warm (June–July 2014) and cold (April 2015) period (Table 1), as fish settlement shows wide seasonal variation^26, 31, 80^. During our study, mean seasonal sea surface temperature ranged from approximately 14 °C (cold period) to 22 °C (warm period). A total of 2101 UVC samples were undertaken. Each sample was a priori attributed to one of 14 habitat types defined according to the main types of substrates present in shallow sublittoral Mediterranean coastal areas, i.e. natural and artificial rocky substrates, soft bottoms and seagrass beds, along with their main ecotones (hereafter named interfaces) (Table 1), according to previous studies on juvenile fish settlement in the Mediterranean Sea^25, 42, 43, 50^. Fishes were recorded among randomly replicated sampling units spread among each treatments: UVCs were done on 2 × 1 m (2 m^2^) quadrates on natural rocky substrates (RS), 5 × 2 m (10 m^2^) belt transects in *C. nodosa* meadows (CY and POCY) and 10 × 1 m (10 m^2^) transects in all other habitats, including *P. oceanica* beds, adapted according to the spatial extent, variability and heterogeneity of habitat characteristics^28, 81^. All fishes smaller than 10 cm in total length (TL) were identified at species or genus level, and their abundance and size (TL, to the nearest cm between 4–10 cm, to 0.5 cm under 4 cm TL) were recorded. In addition, a set of habitat descriptors were recorded in order to verify a posteriori that the sampling units (visually selected) were appropriately classified into meaningful and objectively-defined habitat types. After each fish count, a set of 26 habitat descriptors were recorded in quadrates or when swimming back along transects in order to describe precisely the abiotic and biotic habitat characteristics: depth (m), slope (integer scale from 1 to 6), percent coverage of substrate types (6 types), rugosity classes (4 classes), vegetal types (3 types of seagrasses and 5 types of other macrophytes), and height (cm) of the canopy^26, 28, 32^ (Table 2). For convenience we used the term *Cystoseira* forest although the genus has been recently divided into three^82^. Whatever the genus actually used, *Carpodesmia*, *Treptacantha* or *Cystoseira,* all species display erect arborescent thalli and functionally form a forest^83, 84^.

**Table 1:** Habitat types, codes and number of samples (N) in warm (summer 2014) and cold (spring 2015) periods.

Habitat type	Code	Warm period N	Cold period N
Rocky substrates	RS	328	150
Artificial rocky reefs	AR	20	0
Soft bottoms	SB	271	155
*Cymodocea nodosa* beds	CY	40	5
*Posidonia oceanica* beds	PO	174	90
*Posidonia* barrier reef flat	POBR	75	50
*Posidonia* barrier reef outer slope	POEX	64	50
*Posidonia* barrier reef inner slope	POIN	71	30
Barrier reef lagoon with *Cymodocea*	POCY	51	20
*Posidonia* dead matte	PODM	33	30
Interface *Posidonia*/rocky substrata	IPR	87	50
Interface *Posidonia*/soft bottoms	IPS	126	65
Interface *Posidonia*/dead matte	IPM	25	20
Interface Rocky substrates/soft bottoms	IRS	11	10
TOTAL		1376	725

**Table 2:** Abiotic and biotic habitat descriptors of shallow coastal habitats.

Descriptor	Type	Units, scales or levels
Depth	Continuous	Meters
Slope (integer scale)	Numerical scale of integers from 1 to 6	1 (0–15°); 2 (16–30°); 3 (31–60°); 4 (61–< 90°); 5 (90°); 6 (> 90°)
Substrate (6 types)	% cover for each of 6 types	Rock, blocks, pebbles, gravel, sand, mud
Rugosity (4 types)	% cover of each of 4 types	Low, medium, high, very high
Vegetal coverage: Seagrasses (3 types)	% cover for each of 3 types	*Posidonia oceanica*, dead matte, *Cymodocea nodosa*
Vegetal coverage: Macrophytes (5 types)	% cover for each of 5 types	– *Cystoseira sensus lato* forest (*Carpodesmia brachycarpa, Carpodesmia crinita, Treptacantha barbata, Cystoseira compressa*) – Other arborescent algae (*Halopitys incurva, Spaerococcus coronopifolius*) – Bushland (*Halopteris scoparia, Padina* sp*.,* Dictyotales*, Corallina* sp*., Acetabularia acetabulum, Laurencia* spp.) – Turf/encrusting algae – Wrecked algae
Canopy height	Continuous	Height (cm) of canopy (only for each seagrass or macrophytes types)

For statistical analyses, only the juvenile individuals of the species recorded were considered following literature information, as the size limit between juvenile and adult stages varies among species depending on their maximum size and biology^28, 32, 63^. The species richness was the number of fish species recorded per 10 m^2^ sampling unit and the density was the number of individuals per 10 m^2^ at species (taxa-specific density) or assemblage (total density) level.

### Data analysis

#### Habitat descriptor analysis

A first set of analyses was performed on habitat descriptors for each sample. Multivariate descriptors of habitat were previously standardized (by maximum) and the Euclidean distance was used as a measure of dissimilarity due to the different nature and variation range of the descriptors used^85^. Ordination methods were applied to the distance matrix calculated from this data-frame in order to verify whether samples would be grouped by clusters in accordance with their a priori habitat types. Since a first visual interpretation of ordination bi-plot indicated that samples were in effect grouped by habitat types (see “Results”), we calculated a new matrix of distance between centroids for the grouping factor “Habitat-Station-Period”, which enabled us to display a clearer visual representation using ordination. To represent dissimilarities between samples of habitat descriptor assemblages, we performed a Principal Coordinate Analysis (PCoA) ordinations plot of centroids of descriptor samples of the dummy factor combining station, habitat type and period^86, 87^. Arrows were superimposed onto PCoA bi-plots to represent the Spearman’s rank correlations between biplot axes and habitat descriptors^85^. Complementarily, in order to test whether samples would indeed significantly differ in terms of metrics assemblages as a function of their habitat types, we applied to this last distance matrix a PERMutational multivariate ANalysis Of VAriance (PERMANOVA) using the algorithm developed by Anderson and collaborators^87^. The PERMANOVA model included two factors: (i) “Habitat” was fixed and included 14 levels (Table 1) and (ii) “Period” was fixed and included two levels (warm and cold).

#### Juvenile fish assemblage analysis

We applied the same model (Habitat x Period) in order to test the effect of both factors on the descriptors of the juvenile fish assemblages. We used both univariate (taxa richness, total density—i.e. the sum per replication unit of fish without distinction of their taxa) and multivariate (composition and relative densities of taxa) descriptors as response variables. For each assemblage descriptor (i.e. multivariate taxa densities, richness, total densities and taxa-specific univariate densities), in order to test whether samples would indeed significantly differ in terms of assemblage descriptors as a function of their habitat types and/or period, we applied to each respective distance matrix a PERMutational uni/multivariate ANalysis Of VAriance (PERMANOVA) using the algorithm developed by Anderson et al.^87^. The PERMANOVA model included two factors: (i) “Habitat” was fixed and included 14 levels (Table 1) and (ii) “Period” was fixed and included two levels (warm and cold). For univariate descriptors (richness and total densities), we used the Euclidian distance on untransformed data while for the multivariate assemblage descriptor (relative taxa densities), we used the Modified Gower distance measure on untransformed data, since this distance includes itself of log-transformation (base 2), as suggested by Clarke et al.^85^ and Anderson et al.^87^. Moreover, SIMPER test was used as analysis of species contributions to significant differences between sets of samples^85^. Additionally, for a set of 6 commercially and economically important Sparidae taxa whose juvenile habitats have been particularly described in the past^25^, mean individual sizes (TL, cm) in each habitat and period were compared using t-tests (*Diplodus annularis*, *D. vulgaris*, *D. sargus*, *Oblada melanura*, *Pagellus* spp., *Sarpa salpa*).

Sums of squares (SS) for all PERMANOVA designs were performed as a fully partial analysis (type III). P-values were obtained by 999 permutations of residuals under a reduced model. Monte Carlo P-values were considered when there were not enough possible permutations (< 200). Terms were pooled as suggested by Anderson et al.^87^. Additionally, PERMDISP routine was applied to the same model when needed, in order to compare dispersion range of response variable data around their median values^87^. Tests were considered significant for P-values < 0.05. Multivariate exploratory analyses and both multivariate and univariate inferential tests were performed using the PRIMER 6 software and PERMANOVA + B20 package^86, 87^. Dataset manipulations, basic tests (t-tests) and others graphical visualizations were performed in R Environment^88^ using the library ggplot2^89^.

## Results

### Typology of habitats

**Figure 2:**
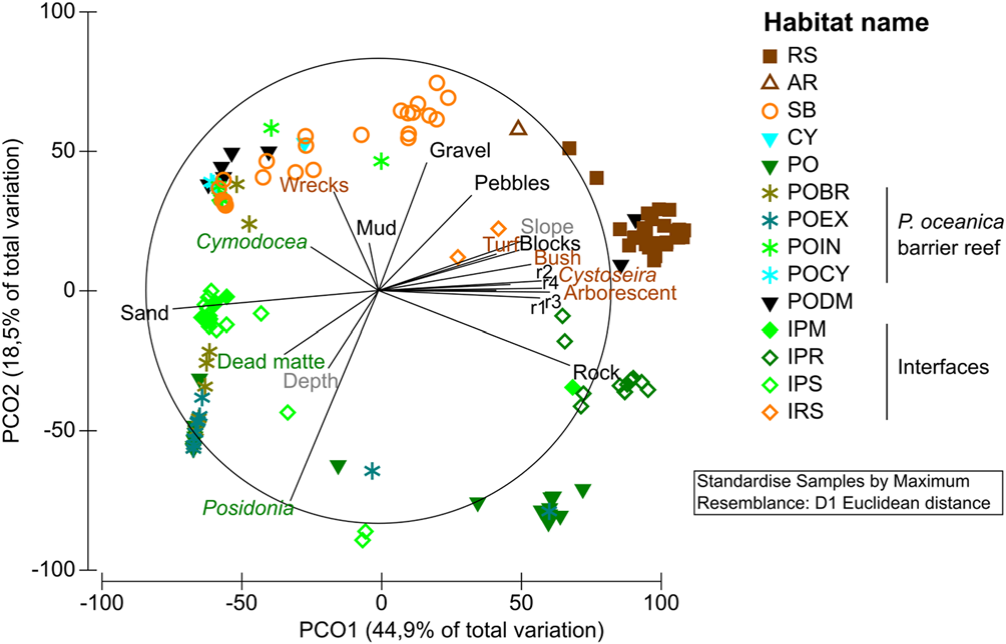
Principal coordinate analysis (PCoA) ordination plot of centroids of habitat descriptor assemblages according to habitat types. Correlation vectors (Spearman) of descriptors are plotted (correlations > 0.2). RS: Rocky substrates; AR: Artificial rocky reefs; SB: soft bottoms; CY: *Cymodocea nodosa* beds; PO: *Posidonia oceanica* beds; POBR: *Posidonia* barrier reef flat; POEX: *Posidonia* barrier reef outer slope; POIN: *Posidonia* barrier reef inner slope; POCY: Barrier reef lagoon with *Cymodocea*; PODM: *Posidonia* dead matte; IPR: Interface *Posidonia*/Rocky substrates; IPS: Interface *Posidonia*/Soft bottoms; IPM: Interface *Posidonia*/Dead matte; IRS: Interface Rocky substrates/Soft bottoms.

Mean habitat descriptors significantly differed between the habitat types a priori defined, while Period and the interaction (Habitat × Period) had no significant effect (PERMANOVA, P-value < 0.001, Table 3, Fig. 2). Among the 91 habitat pairs, 81 pair-wise tests resulted in a significant difference of descriptor assemblage between pairs of habitat types (PERMANOVA, pair-wise tests, all P < 0.05). Such results a posteriori confirmed the validity of the 14 habitat types defined for the fixed factor “habitat”, which remained stable over time whatever the season. These habitat types could be grouped into 3 main categories: rocky substrates, soft bottoms and seagrass beds, with all their interfaces.

**Table 3:** PERMANOVA table of results: comparison of habitat descriptors assemblage per station between habitats and period.

Source of variation	df	MS	Pseudo-F	P (perm)
Habitat type (Ha)	13	72,942	18.367	0.001
Period (Pe)	1	3352.3	0.84415	0.502
Ha × Pe	12	3889	0.9793	0.518
Residuals	130	3971.2		
Total	156			

Table gives degrees of freedom (df), Mean Squares (MS), calculated pseudo-F, and P-values (P). P-values were obtained by 999 permutations of residuals under a reduced model (perm) or through Monte Carlo test (MC, see “Material and methods”).

The first two axes of the PCoA explained 63.4% of the variability of data (81.7% for the first five axes). Rocky substrates (natural and artificial) gathered tightly on the positive part of axis 1 and were correlated not only with rocks and boulders, but also with high slope, high rugosity, and most macrophyte categories. Natural rocky habitats (RS) were characterized in particular by *Cystoseira* forests (21% mean coverage), other arborescent macroalgae (7%; i.e. *Halopitys incurvus*), and bushland communities (49%; i.e. Sphacelariales) (Table S1). Artificial rocky substrates (AR) differed from natural ones by the absence of any type of erect perennial macrophyte forest, but the amount of turf /encrusting algae cover (42%) and bushland (58%). All soft bottoms (SB) clustered on the positive part of axis 2 and were mainly correlated with gravel and floating algal detritus. They were scattered along axis 1 from pebbles (positive part) to sand (negative part) depending on their granulometry. They were also characterized by a low slope and the absence or rarity of algal cover (5% of turf/encrusting algae only). Unlike rocky or soft bottoms, seagrass bed habitats, and particularly those associated with *Posidonia oceanica* (PO), were highly dispersed on the PCoA plane (Fig. 2), in relation with the type of substrate *P. oceanica* is growing on. Stations where *P. oceanica* was growing on rocky substrates clustered on the positive part of axis 1, but on the negative part of axis 1 where it was growing on sandy substrates. Habitats associated with *P. oceanica* barrier reef structure were scattered along axis 2 from high depth and seagrass cover percentage on the barrier reef outer slope (POEX) to a high percentage of sand in the shallow inner slope (POIN) and associated *Cymodocea nodosa* meadows (CY and POCY). POIN was also characterized by the presence of algal wreck, which offered shelter to juvenile fish.

On the plane defined by axes 1 and 2, some PODM stations were gathered with rocky habitats due to a high cover percentage of macrophytes, especially bushland communities (Table S1), but all PODM stations tightly clustered together on the positive part of axis 4, which was correlated with a high dead matte percentage. All interfaces were positioned on the PCoA plan at a logical but well-individualized place testifying to their particular identity: IPR between *Posidonia* and rocky substrates on the positive part of axis 1 and negative part of axis 2, IRS between rocky and soft substrates on the positive parts of both axes, IPS and IPM on the negative part of axis 1, as correlated to high sand and dead matte percentages respectively. Mean values (± SE) of the abiotic and biotic descriptors of the 14 individualized habitat types are given in Table S1, along with their significant seasonal variations. Abiotic descriptors rarely changed with period whatever the habitat and were related to the haphazard position of sampling units, while biotic habitat descriptors presented consistent seasonal variations linked to the biology and growth of macrophytes. Higher cover percentages and canopy height of seagrasses and macroalgae were generally recorded in warm rather than in cold period, except for turf/encrusting and wrecked algae, which increased the three-dimensional structure of these habitats (Table S1). However, differences were statistically significant only in a few habitats due to the high variance of data.

### Juvenile fish assemblages

**Figure 3:**
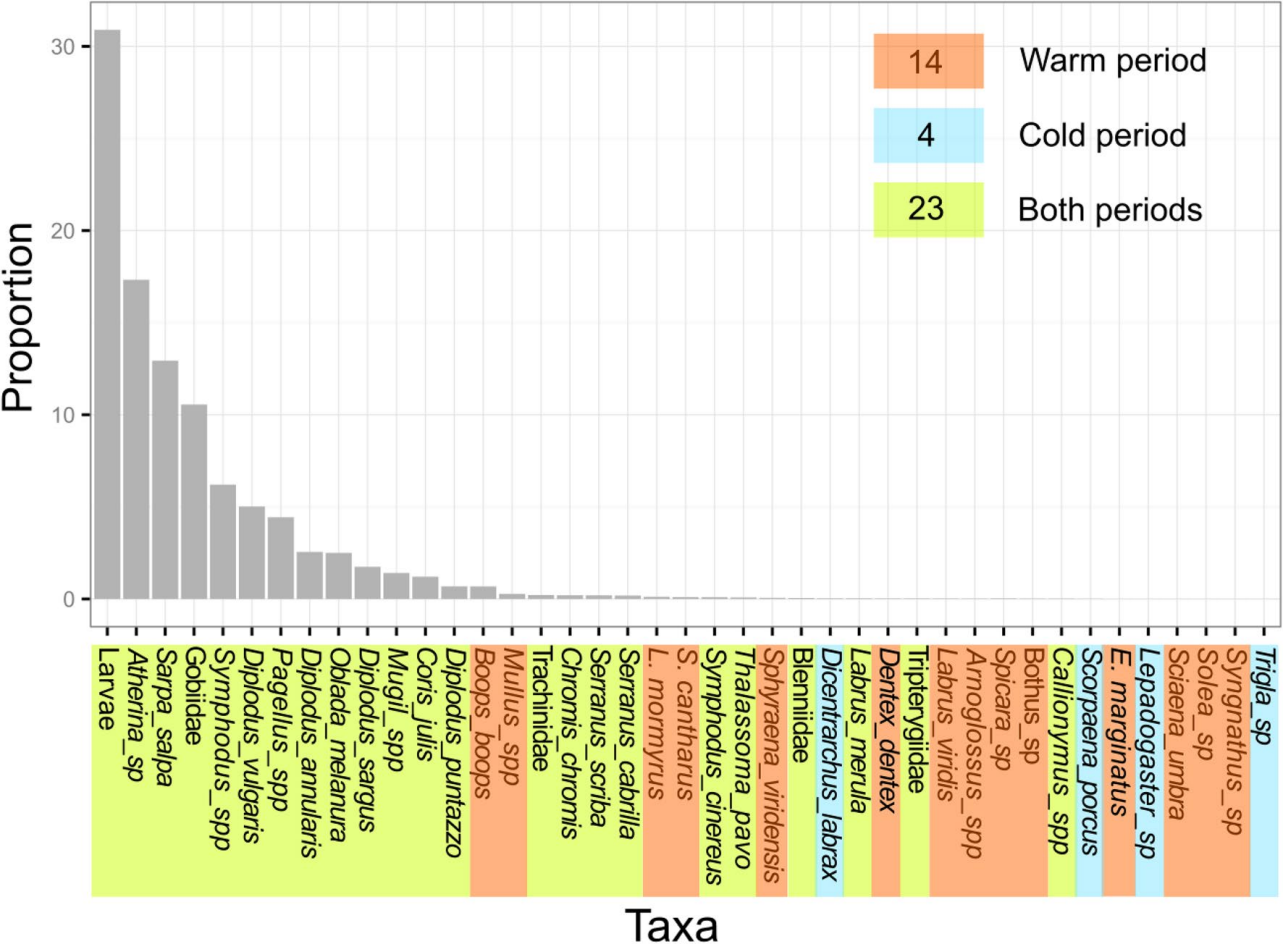
Proportion of each observed taxa in the total abundance of juvenile fishes recorded in all samples and habitats combined; *E.* = *Epinephelus*, *L.* = *Lithognathus, S.* = *Spondyliosoma*. Period(s) of observation of each taxa and total number of taxa observed per period are indicated with colored boxes.

A total of 526,014 juvenile individuals, belonging to 57 different fish species/taxa and 22 families were recorded (Table S2). As small juveniles of particular genera such as *Symphodus,* or families such as blenniids and gobiids, were difficult to identify precisely underwater, they were grouped into 41 taxa for analysis. A higher total species richness of juvenile fish was recorded during the warm (37 taxa, n = 1 376) than the cold (27 taxa, n = 725) period and differed among habitats (Table 4). The highest total number of taxa was recorded on soft bottoms (25 taxa, n = 426), followed by rocky substrates and *Posidonia* beds (22 taxa each, n = 428), while the lowest number of juvenile fish species was observed in *Cymodocea* beds (6 taxa, n = 116). The most abundant taxa included by decreasing order of importance unidentified larvae, *Atherina* sp., *Sarpa salpa*, Gobiidae, *Symphodus* spp., *Diplodus vulgaris*, *Pagellus* spp., *Diplodus annularis*, *Oblada melanura* and *Diplodus sargus* (Fig. 3). They were observed in most habitats (from 7 habitats for *Pagellus* spp. to 14 for *D. vulgaris*), while 13 taxa were recorded in only one habitat type, generally with low abundance (Table S3). Four taxa were only recorded on rocky substrates (*Boops boops, Epinephelus marginatus*, *Thalassoma pavo* and Tripterygiidae) and four on soft bottoms (*Arnoglossus* spp., *Bothus* sp., *Solea* sp. and Trachinidae). Most species (23 spp.) were observed at both periods, and a higher number were recorded only in warm than only in cold periods (14 spp. *vs* 4 spp., respectively) (Fig. 3).

**Table 4:** Total number of fish taxa recorded in the different juvenile habitats of shallow coastal areas, and during the warm (summer 2014) and cold (spring 2015) periods.

Habitat type	Total	Warm period	Cold period
RS	22	22	15
AR	14	14	–
SB	25	22	15
CY	6	6	2
PO	22	21	10
POBR	15	14	8
POEX	16	15	9
POIN	14	13	6
POCY	16	15	3
PODM	12	10	4
IPR	21	20	8
IPS	21	21	12
IPM	11	8	6
IRS	8	7	3
TOTAL	41	37	27

**Figure 4:**
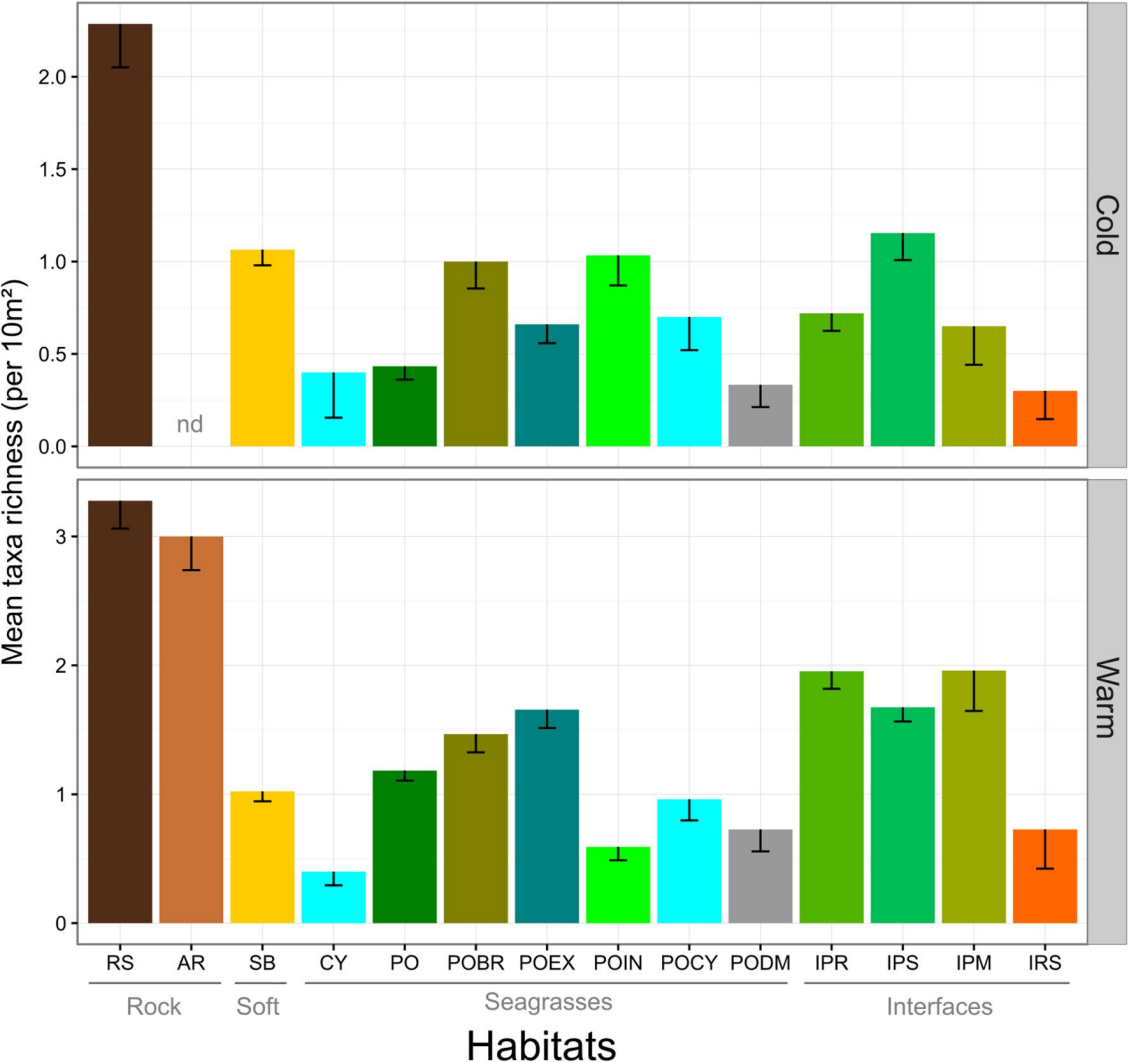
Mean taxa richness (± SE) of juvenile fishes per 10 m^2^ in shallow coastal juvenile habitats for both periods (Cold and Warm). RS: Rocky substrates; AR: Artificial rocky reefs; SB: soft bottoms; CY: *Cymodocea* beds; PO: *Posidonia oceanica* beds; POBR: *Posidonia* barrier reef flat; POEX: *Posidonia* barrier reef outer slope; POIN: *Posidonia* barrier reef inner slope; POCY: Barrier reef lagoon with *Cymodocea*; PODM: *Posidonia* dead matte; IPR: Interface *Posidonia*/Rocky substrates; IPS: Interface *Posidonia*/Soft bottoms; IPM: Interface *Posidonia*/Dead matte; IRS: Interface Rocky substrates/Soft bottoms. Main habitat categories are indicated in grey.

Mean species richness per sample varied from 0.25 to 3 taxa per 10 m^2^ and differed according habitats and periods. The significant interaction of the two factors indicated that between-habitat variability differed between seasons (PERMANOVA, F = 6.506, P < 0.001, Table 5). Mean species richness was highest on natural and artificial rocky substrates and lowest in *Cymodocea* beds (Fig. 4). Interfaces *Posidonia*/other habitats (IPR, IPS and IPM) presented a higher mean species richness than the different habitats of *P. oceanica* bed and barrier reef, demonstrating the particular importance of ecotones for juvenile fishes. The mean species richness tended to be higher during the warm than the cold period, in all but POIN habitat, where a lower mean species richness was recorded in summer (Fig. 4).

**Table 5:** PERMANOVA table of results: comparison of taxa richness of juvenile assemblages between habitats and periods.

Source	df	MS	Pseudo-F	P(perm)
Habitat type (Ha)	13	28.253	22.632	0.001
Period (Pe)	1	53.082	42.52	0.001
HaxPe	12	7.4477	5.9658	0.001
Res	1689	1.2484		
Total	1715			

Table gives degrees of freedom (df), Mean Squares (MS), calculated pseudo-F, and P-values (P). P-values were obtained by 999 permutations of residuals under a reduced model (perm) or through Monte Carlo test (MC, see “Material and methods”).

**Figure 5:**
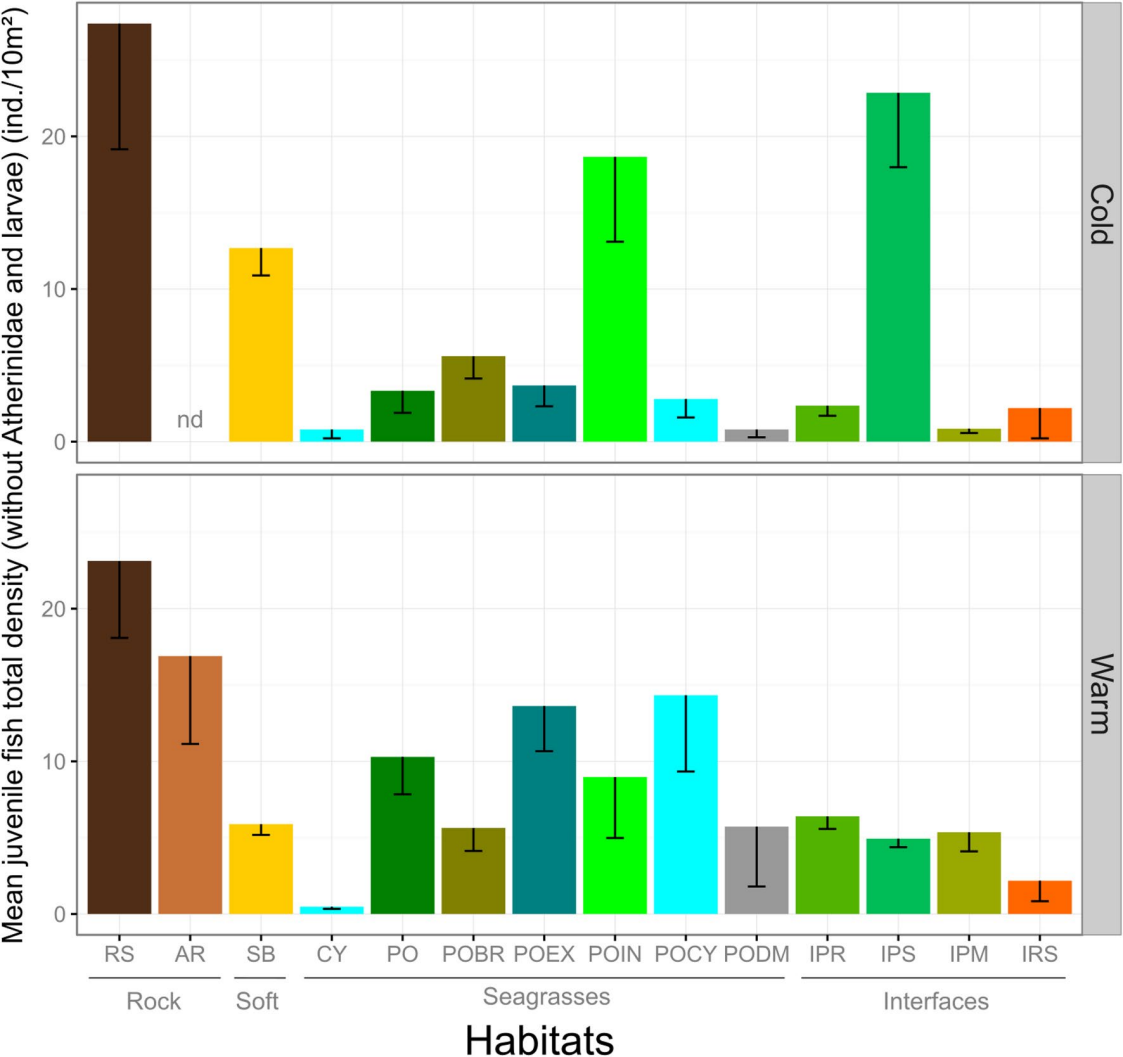
Mean (± SE) total density (without Atherinidae and larvae) of juvenile fishes among habitats for both periods (Cold and Warm). RS: Rocky substrates; AR: Artificial rocky reefs; SB: soft bottoms; CY: *Cymodocea* beds; PO: *Posidonia oceanica* beds; POBR: *Posidonia* barrier reef flat; POEX: *Posidonia* barrier reef outer slope; POIN: *Posidonia* barrier reef inner slope; POCY: Barrier reef lagoon with *Cymodocea*; PODM: *Posidonia* dead matte; IPR: Interface *Posidonia*/Rocky substrates; IPS: Interface *Posidonia*/Soft bottoms; IPM: Interface *Posidonia*/Dead matte; IRS: Interface Rocky substrates/Soft bottoms. Main habitat categories are indicated in grey; “nd” = no data available.

As schools of larvae and *Atherina* sp. could be numerous and haphazardly dispersed in space and time, they might mask the effect of period or habitat on relative abundance. *Atherina* sp. were more abundant during the warm period and were present in 12 habitats, while undetermined larvae were observed in higher abundance during the cold period and present in 13 habitat types. They were thus excluded from the analysis of juvenile abundance to obtain clearer patterns. The total density of juvenile fishes also varied significantly between habitats and periods, with different patterns according to the period (PERMANOVA, F = 6.028, P < 0.001; Fig. 5; Table 6). In most habitats, except POIN and IPS, juvenile fish abundance was higher during the warm season, especially in *Posidonia* seagrass beds and barrier reef outer slope (POEX) and lagoon (POCY). The mean abundance of juvenile fish, all habitats combined, did not differ significantly with period (Table 6), reaching 8.48 ± 0.69 individuals per 10 m^2^ during the warm period and 9.59 ± 0.94 individuals per 10 m^2^ during the cold period.

**Table 6:** PERMANOVA table of results: comparison of total juvenile density between habitats and periods.

Source	df	MS	Pseudo-F	P (perm)
Habitat type (Ha)	13	3002.7	6.0563	0.001
Period (Pe)	1	9.7155	0.019596	0.878
HaxPe	12	2364.2	4.7686	0.001
Res	1689	495.79		
Total	1715			

Table gives degrees of freedom (df), Mean Squares (MS), calculated pseudo-F, and P-values (P). P-values were obtained by 999 permutations of residuals under a reduced model (perm) or through Monte Carlo test (MC, see “Material and methods”). Atherinidae and larvae have been removed for a clearer representation.

### Variability of juvenile fish assemblages in habitats

**Figure 6:**
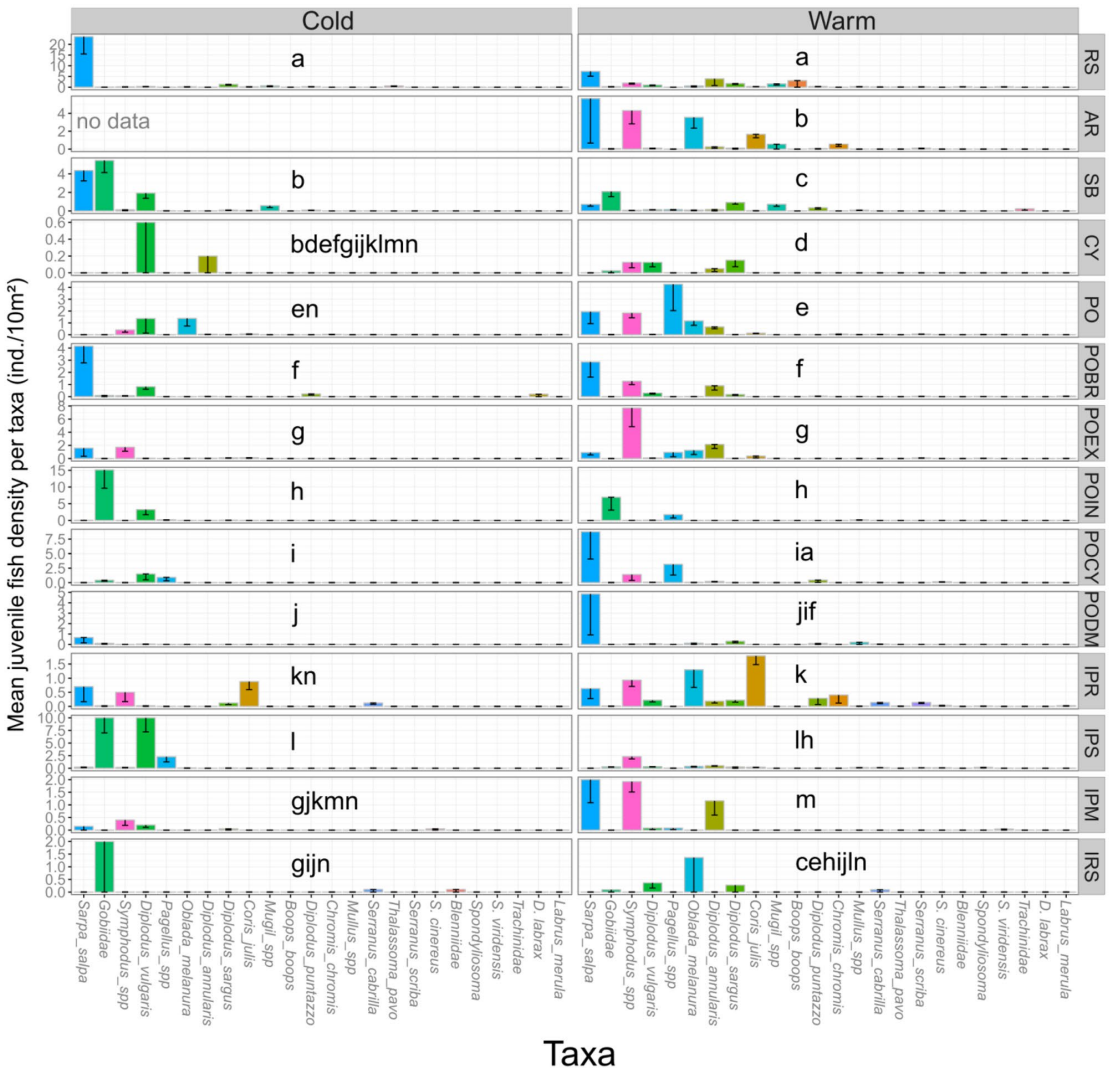
Mean (± SE) juvenile density of each taxa in juvenile habitats for both periods (Cold and Warm). Atherinidae and larvae, as well as the 15 least abundant taxa, were removed for a clearer representation. Note that vertical axes display different scales. *S. cinereus* = *Symphodus cinereus*; *S. viridensis* = *Sphyraena viridensis*; *D. labrax* = *Dicentrarchus labrax*; Details of taxa are given in Table S2. For each period, comparisons of juvenile fish assemblages between juvenile habitats (pairwise tests results) are given (treatments that share at least one lower case character do not significantly differ).

**Figure 7:**
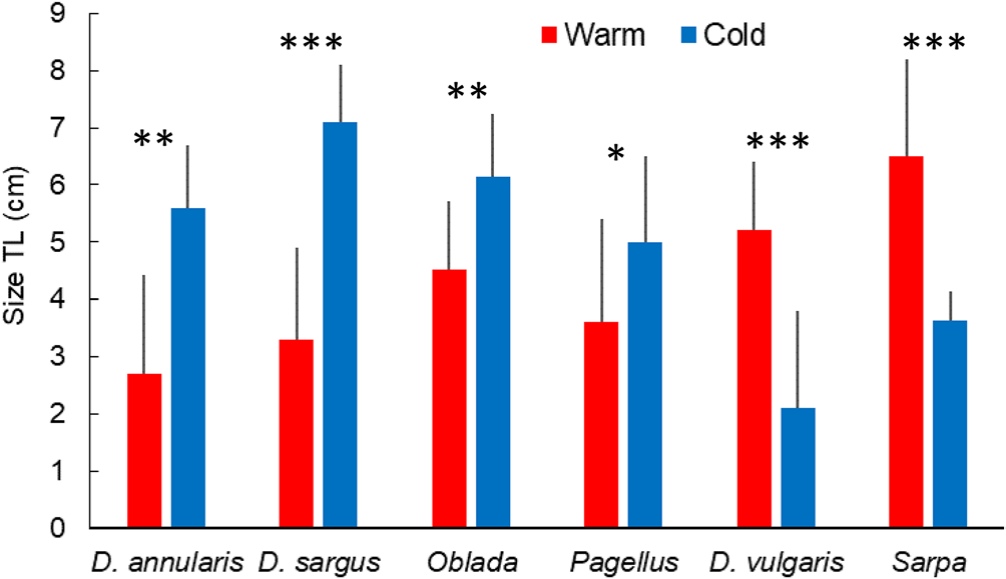
Mean size (TL cm ± SD) of some fish species juveniles settling in Mediterranean shallow coastal habitats. *D.* = *Diplodus*; *Oblada* = *Oblada melanura*; *Pagellus* = *Pagellus* spp.; *Sarpa* = *Sarpa salpa*. Warm: warm period (summer 2014); Cold: cold period (spring 2015). Results of t-test for difference in mean size of a given species according to period: *P < 0.05; **P < 0.01; ***P < 0.001.

The assemblage composition of juvenile fishes in terms of relative taxa-specific densities significantly differed between periods, and these differences were specific to each habitat as a significant interaction between the two factors was evidenced (PERMANOVA, F = 7.743, P < 0.001; Fig. 6; Table 7). Seasonal differences in juvenile assemblage occurred in most habitat types, except CY, POCY and IRS. Reciprocally, assemblage composition of juvenile fishes differed between habitats, and these differences were specific to each period. These assemblage differences between treatments (habitat type × period) were due both to differences of mean assemblage composition (i.e. centroids) and of assemblage dispersion (i.e. heterogeneity) (PERMDISP test, F = 11.088, P < 0.001). Overall, taxa mainly responsible of assemblage dissimilarities between periods (SIMPER test, Average dissimilarity = 94.12) were Gobiidae (20.9%), *Sympodus* spp. (12%), *Sarpa salpa* (11.6%), *Diplodus vulgaris* (11%), and in a lesser extent *D. sargus* (6.3%) and *D. annularis* (6.2%). On rocky substrates (RS), no difference in juvenile fish assemblage according to the relative importance of macroalgal cover types (*Cystoseira* forest, bushland or turf/encrusting) was observed (PERMANOVA, F = 0.87, P = 0.531). While the assemblage differed with period (pair-wise test, t = 1.594, P = 0.025), *Sarpa salpa* was the most abundant species on RS in both periods, followed by *Diplodus annularis*, *Boops boops* and *Symphodus* spp. in the warm period, and by *D. sargus*, *Mugil* spp., and *Thalassoma pavo* in the cold period (Fig. 6). *S. salpa* also dominated in abundance on artificial structures (AR) in warm period, with *Symphodus* spp., *Oblada melanura* and *Coris julis*. On soft bottoms (SB), Gobiidae followed by *S. salpa* dominated in both periods, while the assemblage statistically differed (t = 3.406, P < 0.001). During the warm period, *Mugil* spp., *D. sargus* and *D. puntazzo* were also abundant on SB and particularly associated with high percentages of pebbles and gravel, while *Pagellus* spp., *Lithognathus mormyrus*, *Mullus* spp., Trachinidae, *Bothus* sp. and *Solea* sp. were more associated with sand. During the cold period, *D. vulgaris* was particularly abundant on SB and mainly associated with pebbles and gravel. In *Posidonia* beds (PO), the juvenile fish assemblage slightly differed according to the type of substrate *P. oceanica* was growing on. Higher abundances of *D. annularis* and *Symphodus* spp. were recorded when the seagrass was growing on sand, and of *O. melanura* and *S. salpa* when growing on rocky substrates. A seasonal variation was observed in PO (t = 3.298, P < 0.001) with high abundances of *Pagellus* spp., *S. salpa*, *Symphodus* spp. during the warm period, and the dominance of *D. vulgaris* in the cold period. *Oblada melanura* remained abundant all the year in PO. In the different barrier reef habitats, the juvenile assemblage differed with periods, while some species dominated in both periods, such as *S. salpa* in POBR (t = 2.335, P < 0.001) and PODM (t = 2.048, P = 0.007), *Symphodus* spp. in POEX (t = 2.651, P < 0.001), and Gobiidae plus *Pagellus* spp. in POIN (t = 3.174, P < 0.001) (Fig. 6). Each type of interface was dominated by the abundance of some species, and presented seasonal variations, except IRS (t = 1.101, P = 0.289) dominated by Gobiidae, *Serranus cabrilla* and *O. melanura*. At IPR, *C. julis* was the dominant species in both periods, but with a far higher abundance in the warm period (t = 2.083, P = 0.005) followed by *O. melanura* and *Symphodus* spp., and by *S. salpa* and *S. cabrilla* in the cold period. At IPS, the juvenile assemblage was dominated by *Symphodus* spp., *D. annularis, and O. melanura* during the warm period, and by Gobiidae, *D. vulgaris* and *Pagellus* spp. in the cold period (t = 1.608, P = 0.038). At IPM, the dominant species was *S. salpa* in the warm period, and *Symphodus* spp. in the cold period (t = 3.331, P < 0.001). Noteworthy was the higher presence of predators (*Serranus cabrilla*, *S. scriba*, *Scorpaena porcus*, *Dentex dentex* and *Labrus viridis*) in interface habitats (Fig. 6). In *Cymodocea* meadows (CY and POCY) no statistical difference was observed with period (t = 1.221, P = 0.212 and t = 1.297, P = 0.162, respectively) and the assemblage was dominated by *D. vulgaris* in CY and by *Pagellus* spp., *D. vulgaris* and Gobiidae in POCY. Thus, several species dominated in different juvenile habitats in both periods. However, the mean size of juvenile fishes differed between periods for some of the most abundant species (Fig. 7). Smaller-sized juveniles were observed during the warm period for *D. annularis*, *D. sargus*, *O. melanura* and *Pagellus* spp., and during the cold period for *D. vulgaris* and *S. salpa.*

**Table 7:** PERMANOVA table of results: comparison of multivariate assemblage of juvenile density between habitats and periods.

Source	df	MS	Pseudo-F	P(perm)
Habitat type (Ha)	13	17.591	10.738	0.001
Period (Pe)	1	19.628	11.982	0.001
HaxPe	12	7.8712	4.8049	0.001
Res	1689	1.6382		
Total	1715			

Table gives degrees of freedom (df), Mean Squares (MS), calculated pseudo-F, and P-values (P). P-values were obtained by 999 permutations of residuals under a reduced model (perm) or through Monte Carlo test (MC, see “Material and methods”).

## Discussion

Higher juvenile fish mean species richness and abundance occurred during the warm in comparison with the cold period. Habitats hosting the richest and most abundant juvenile fish assemblages were the natural rocky substrates and the interfaces between *Posidonia* beds and the other habitats. Juvenile fishes were recorded in all habitat types, although juvenile fish assemblage composition differed among habitat types and between periods. The most abundant fish species were *Atherina* sp., *Sarpa salpa*, Gobiidae, *Symphodus* spp., *Pagellus* spp. and several *Diplodus* species, which colonized 7 up to 14 different habitat types (depending on taxa) during their juvenile life. Most species settled in one or a few specific habitats but rapidly colonized adjacent habitats when growing.

### Habitats used by juvenile fish in Mediterranean shallow coastal zones

All the sites surveyed hosted juveniles in both warm and cold period, highlighting the crucial functional role of the very shallow coastal bottoms as fish nurseries. In contrast to findings based on the habitat- and species-centered approaches, in the present study juvenile fish assemblages were recorded in all types of habitats encountered in Mediterranean shallow coastal zone. A total of 14 different habitat types were characterized, which could be grouped into three broad categories, rocky substrates (natural RS and artificial AR), sedimentary bottoms (SB) with all levels of granulometry, and seagrass beds including *Cymodocea nodosa* and *Posidonia oceanica* meadows (CY, PO, PODM) (Table 1). The ecotones or interfaces between the three broad habitat categories (IPM, IPR, IPS and IRS), were individualized as particular habitat types. We evidenced that if the structural characteristics of habitat types did not vary with period, the biological characteristics did vary with higher cover percentages and canopy height of seagrasses and macroalgae in the warm period (Table S1).

The presence of juveniles was evidenced in every type of sampled habitat. While the habitat types were well individualized, it appeared that one third of fish species occupied more than 7 habitat types when juveniles and were the most abundant species (34.1% of total species richness and 95.2% of total abundance), while the one third of species characteristic of only one habitat type were rather rare (31.7% of total species richness and 1.1% of total abundance). If atherinids and larvae were excluded, the relative abundance of the common and restricted species remained similar (90.8% and 2.0% respectively). This means that several habitats are used and necessary for the ontogenetic development of most species when juveniles, including different common commercial seabreams (*Diplodus*, *Sarpa*, *Pagellus*). The rapid ontogenetic changes in morphology, diet and behavior of juvenile fishes^28, 55, 63^ result in a rapid increase of their spatial ecological niche^20, 48^. Furthermore, the abundance of the species considered as rare in this study was probably underestimated as difficult to record by UVC, being either buried in sand such as flatfishes (*Arnoglossus*, *Bothus*, *Solea*) or hidden in rocky crevices such as scorpionfishes (*Scorpaena*) or groupers (*Epinephelus*).

Habitat and seascape tri-dimensional structure can be qualified by its heterogeneity and complexity^90, 91^. Generally high quality habitats for juvenile fishes are recognized to be associated with high degrees of three-dimensional structuration^92^, in terms of both complexity^51, 53, 93, 94^ and/or heterogeneity^42^. Natural rocky habitats (RS) presented a high structural and biological complexity due to different macrophytes assemblages, and indeed supported the highest mean species richness and abundance of juvenile fishes in the two sampling period (cold and warm). However, SB while presenting a lower structural complexity than RS or PO, supported the highest total species richness (24 spp.) of juvenile fish owing to an intermixed diversity of granulometry and the ability it offers to the juveniles to blend in with the bottom. It was also evidenced that interfaces represented highly favorable habitats for juveniles in terms of both species richness and abundance (Figs. 4 and 5). Ecotones (i.e. interfaces) have long been known as increasing the diversity of fish communities^95^ and their role in the dynamics of rocky fish assemblages associated with *Cystoseira* forests was recently studied in the Mediterranean Sea^52, 55^. Interfaces, particularly between *Posidonia* beds and adjacent habitats (IPS, IPR, IPM), harbored a high number of juveniles of piscivorous fishes such as *Serranus cabrilla*, *Scorpaena porcus*, *Dentex dentex*, and *Labrus viridis*, which found here a suitable place for predation. This is consistent with previous studies highlighting the suitability of ecotones for various predation strategies (ambush, stalk-attack, etc.)^55^ and for avoidance of predators by their juvenile prey^94^. In the case of the *Posidonia oceanica* barrier reef complex, we provided evidence that different juvenile fish assemblages were associated with the different parts of the barrier reefs including reef flat, slopes and lagoon (POBR, POEX, POIN, and POCY). The barrier reef complex is by nature a juxtaposition of various habitat patches along with their interfaces; this habitat diversity allows various species to find suitable juvenile habitats, as illustrated in the case of tropical reef habitat systems^96, 97^.

### Temporal succession of nursery use by juvenile fishes

We observed that juvenile fish assemblages presented higher species richness and abundance during the warm than the cold period in most habitat types. The specific composition of the juvenile assemblage is directly linked to the reproductive cycle of coastal fish species. Juvenile fishes settling in coastal nurseries during the warm period were issued from adults reproducing in spring or early summer, as the duration of larval life for most Mediterranean coastal fish species ranges from 2 to 6 weeks^15, 66, 98^. Those arriving during the cold period resulted from the reproduction of adults in late summer, autumn and winter. We observed in effect the smallest *D. annularis* and *D. sargus* juveniles during the warm period and the smallest *D. vulgaris* and *S. salpa* during the cold period (Fig. 7), following a well-known temporal succession of juvenile fish species in coastal nurseries^26, 31, 33, 62, 72, 99^. If juvenile fishes settle sometimes in highly specific habitats, they rapidly expand their home range when growing and increasing their swimming capacities, colonizing deeper or adjacent habitats^20^, and leaving settlement sites available for the successive arrival of fish post-larvae^60, 62, 99^. By performing such ontogenetic habitat shifts as they grow, juveniles tend to switch to using the habitat best fitting their needs in terms of food *versus* refuge against predation availability (the “habitat quality” ratio)^19^. We observed in effect the presence of juveniles of > 34% fish species in more than half of the habitat types individualized indicating that they were used by fishes at various stages of their juvenile life. Thus, for most species, the presence of a mosaic of different habitats is essential for the success of juvenile fish recruitment^51, 52^.

### Importance of both local habitat characteristics and large-scale environmental conditions

However, the higher abundance of juveniles in seagrass bed habitats and rocky substrates during the warm period could be related to the greater protection and food resources provided by the greater canopy height of *Posidonia oceanica* and macroalgae communities^50, 51, 53^. The role of highly complex *Cystoseira* forest canopies with regard to the composition of juvenile fish assemblage was well studied by Cheminée et al.^50, 51^, Cuadros et al.^52^ and Hinz et al.^53^, who demonstrated that *Symphodus* spp., *Labrus* spp. and *Serranus* spp. were more abundant in dense complex forests, while *C. julis* and *T. pavo* preferred less complex patches of bare substratum located at the edges of the forests. In our study, *Cystoseira* forests where not extensive enough to form large, dense forests such as those studied by these authors in Corsica and the Balearic Islands, but were mixed with patches of other erect macroalgae, bushland and turf algae. This was the reason why no correlation was found between the cover percentages of these macrophyte assemblages and the composition of the juvenile fish assemblages on the coasts of Western Provence (authors’ unpublished data). The decline and scarcity of erect macroalgae forests (notably *Cystoseira* spp.) has been documented in the last two decades in different parts of the Mediterranean coasts^100–103^. Decline of forests occurs through ecosystem shifts resulting from cascading effects from a wide array of anthropogenic pressures^104–106^. Such profound transformations of the seascape is known to have damaging effects on habitats’ nursery role^28, 50, 52, 54^. Therefore, in those altered areas, the habitat quality available nowadays for juvenile fishes is probably several orders of magnitude below what it could be^51^. This highlights the importance of preserving what is left of the nursery function of coastal areas. In order to preserve this function, habitats should in particular be protected against destruction but also against any kind of transformation of their tri-dimensional structure and composition.

On the other hand, juvenile fishes' abundance^62^, growth^107^ and mortality^108^ vary considerably in space and time due to natural stochastic processes linked to both environmental conditions (currents, winds, hydrological parameters)^49, 59^ and the success of adult reproduction^109^, being high or low at one place from one year to another. The same nursery site can therefore perform as a ‘good’ nursery site one year and not the following one^62^. Similarly, the same habitat can perform as a ‘good’ nursery at a given site but not in another location^64^. Thus, the success of fish nurseries does not depend only on the local characteristics of habitats but also on large scale environmental phenomena that determine the initial intensity and trajectory of the flux of fish larvae^80, 110^.

### Importance of the mosaic of habitats for coastal fishes

We provided evidence that the most abundant fish species in Mediterranean shallow coastal areas used several habitat types as nurseries whatever the period, even if juvenile fish assemblages presented specificities in composition and relative abundance of species in each habitat type (Fig. 6). It could be thus claimed that all habitat types present an actual potential as nursery sites for Mediterranean coastal fishes, and that a diversified mosaic of habitats would be the most efficient way to promote high and successful juvenile fish recruitment by providing contiguous shelters and food resources for the different stages of fishes' juvenile life. These results are in agreement with the seascape nursery approach developed by Nagelkerken et al.^111^, which conceptualizes the role of functionally connected multiple mosaics of habitats for fish nursery management^64, 112–114^.

The effective management of coastal zones often consists in a non-fair trade-off between destructive or impacting human activities (harbor and marina constructions, sewage and industrial outflow, etc.) and efforts for environmental protection mainly represented by the implementation of marine protected areas^115, 116^. A pernicious consequence of the current awareness of the economic value of ecosystems and their ecological services to human populations^117^ often resides in a hierarchical view of ecosystems or habitats depending on the intended goals of users. For example, in the Mediterranean Sea *Posidonia oceanica* seagrass beds benefit from a particular protection status^118^, and coralligenous reefs merit special attention^119^. The results of the present study evidence the importance of all types of shallow coastal habitats as nursery sites for Mediterranean fishes whatever the period considered, which strongly supports the general seascape nursery theory of Nagelkerken et al.^111^ and the views of Cheminée et al.^113^ and Cuadros et al.^52^ for the Mediterranean Sea, regarding the importance of protecting the mosaic of habitats for the good health and functioning of coastal ecosystems. The preservation of a mosaic of habitats along the coast, notably in very shallow waters, therefore constitutes the best way to preserve both fish biodiversity and fishery resources. This study highlights that conservation in France is often disconnected from biological reality with, except in a few Marine Parks (which represent a small portion of coastline^120^), most of the shallow habitats not taken into account in any protection plan. The application of the UE Marine Strategy Framework Directive currently promotes the identification of key marine habitats, which is under process notably in France. Our study supports the idea that a greater number of coastal habitats should be considered for protection, compared to the few currently protected by previous directives.

## Supplementary Information

Supplementary Information.

## References

[1] Costanza R (2014). Changes in the global value of ecosystem services. Glob. Environ. Change.

[2] Costanza R (1997). The value of the world’s ecosystem services and natural capital. Nature.

[3] Halpern BS (2008). A global map of human impact on marine ecosystems. Science.

[4] Lindeboom H, Field JG (2002). The coastal zone: An ecosystem under pressure. Oceans 2020: Science Trends and the Challenge of Sustainability.

[5] Airoldi L, Balata D, Beck MW (2008). The Gray Zone: Relationships between habitat loss and marine diversity and their applications in conservation. J. Exp. Mar. Biol. Ecol..

[6] Islam S, Tanaka M (2004). Impacts of pollution on coastal and marine ecosystems including coastal and marine fisheries and approach for management: A review and synthesis. Mar. Pollut. Bull..

[7] Vikas M, Dwarakish GS (2015). Coastal pollution: A review. Aquat. Procedia.

[8] Blaber SJM (2000). Effects of fishing on the structure and functioning of estuarine and nearshore ecosystems. ICES J. Mar. Sci..

[9] Hussein C (2011). Assessing the impact of artisanal and recreational fishing and protection on a white seabream (*Diplodus sargus sargus*) population in the north-western Mediterranean Sea using a simulation model. Part 1: Parameterization and simulations. Fish. Res..

[10] Hawkins AD, Popper AN (2017). A sound approach to assessing the impact of underwater noise on marine fishes and invertebrates. ICES J. Mar. Sci..

[11] Beck MW (2001). The identification, conservation, and management of estuarine and marine nurseries for fish and invertebrates. Bioscience.

[12] Carr MH (1991). Habitat selection and recruitment of an assemblage of temperate zone reef fishes. J. Exp. Mar. Biol. Ecol..

[13] Sheaves M, Baker R, Johnston R (2006). Marine nurseries and effective juvenile habitats: an alternative view. Mar. Ecol. Prog. Ser..

[14] Leis JM (2006). Are larvae of demersal fishes plankton or nekton?. Adv. Mar. Biol..

[15] Raventos N, Macpherson E (2001). Planktonic larval duration and settlement marks on the otoliths of Mediterranean littoral fishes. Mar. Biol..

[16] Di Franco A (2015). Dispersal of larval and juvenile seabream: Implications for Mediterranean marine protected areas. Biol. Conserv..

[17] Di Franco A (2012). Assessing dispersal patterns of fish propagules from an effective Mediterranean marine protected area. PLoS ONE.

[18] Di Franco A, Guidetti P (2011). Patterns of variability in early-life traits of fishes depend on spatial scale of analysis. Biol. Lett..

[19] Dahlgren CP, Eggleston DB (2000). Ecological processes underlying ontogenetic habitat shifts in a coral reef fish. Ecology.

[20] Macpherson E (1998). Ontogenetic shifts in habitat use and aggregation in juvenile sparid fishes. J. Exp. Mar. Biol. Ecol..

[21] Dahlgren CP (2006). Marine nurseries and effective juvenile habitats: Concepts and applications. Mar. Ecol. Prog. Ser..

[22] Jennings S, Blanchard JL (2004). Fish abundance with no fishing: predictions based on macroecological theory. J. Anim. Ecol..

[23] Jones GP (1990). The importance of recruitment to the dynamics of a coral reef fish population. Ecology.

[24] Sheaves M, Baker R, Nagelkerken I, Connolly RM (2015). True value of estuarine and coastal nurseries for fish: Incorporating complexity and dynamics. Estuaries Coasts.

[25] Harmelin-Vivien ML, Harmelin JG, Leboulleux V (1995). Microhabitat requirements for settlement of juvenile Sparid fishes on Mediterranean rocky shores. Hydrobiologia.

[26] Garcia-Rubies A, Macpherson E (1995). Substrate use and temporal pattern of recruitment in juvenile fishes of the Mediterranean littoral. Mar. Biol..

[27] Vigliola, L. Contrôle et régulation du recrutement des Sparidés (Poissons, Téléostéens) en Méditerranée : importance des processus pré- et post-installation benthique. *Thèse Doct Sci Univ Aix-Marseille II Marseille*. (1998).

[28] Cheminée A (2012). Ecological Functions, Transformations and Management of Infralittoral Rocky Habitats from the North-Western Mediterranean: The Case of Fish (Teleostei) Nursery Habitats.

[29] Macpherson E, Zika U (1999). Temporal and spatial variability of settlement success and recruitment level in three blennoid fishes in the northwestern Mediterranean. Mar. Ecol. Prog. Ser..

[30] Heck K, Hays G, Orth R (2003). Critical evaluation of the nursery role hypothesis for seagrass meadows. Mar. Ecol. Prog. Ser..

[31] Félix-Hackradt FC, Hackradt CW, Treviño-Otón J, Pérez-Ruzafa A, García-Charton JA (2013). Temporal patterns of settlement, recruitment and post-settlement losses in a rocky reef fish assemblage in the South-Western Mediterranean Sea. Mar. Biol..

[32] Cuadros A (2015). Settlement and Post-Settlement Processes of Mediterranean Littoral Fishes: Influence of Seascape Attributes and Environmental Conditions at Different Spatial Scales.

[33] Bussotti S, Guidetti P (2011). Timing and habitat preferences for settlement of juvenile fishes in the marine protected area of torre guaceto (south-eastern Italy, Adriatic Sea). Ital. J. Zool..

[34] Bariche M, Letourneur Y, Harmelin-Vivien M (2004). Temporal fluctuations and settlement patterns of native and lessepsian herbivorous fishes on the lebanese coast (Eastern Mediterranean). Environ. Biol. Fishes.

[35] Mosconi P, Chauvet C (1990). Growth spatio-temporal variability of juveniles of sea-bream (*Sparus aurata*) between lagoonal and sea areas in the south of Lion’s Gulf. Vie Milieu Paris.

[36] Verdiell-Cubedo D, Oliva-Paterna FJ, Ruiz-Navarro A, Torralva M (2013). Assessing the nursery role for marine fish species in a hypersaline coastal lagoon (Mar Menor, Mediterranean Sea). Mar. Biol. Res..

[37] Letourneur Y, Darnaude A, Salen-Picard C, Harmelin-vivien M (2001). Spatial and temporal variations of fish assemblages in a shallow Mediterranean soft-bottom area (Gulf of Fos, France). Oceanol. Acta.

[38] Le Pape O (2013). Sources of organic matter for flatfish juveniles in coastal and estuarine nursery grounds: A meta-analysis for the common sole (*Solea solea*) in contrasted systems of Western Europe. J. Sea Res..

[39] Guidetti P, Bussotti S (1997). Recruitment of Diplodus annularis and Spondyliosoma cantharus (Sparidae) in shallow seagrass beds along the Italian coasts (Mediterranean Sea). Mar. Life.

[40] Guidetti P, Bussotti S (2000). Fish fauna of a mixed meadow composed by the seagrasses Cymodocea nodosa and Zostera noltii in the Western Mediterranean. Oceanol. Acta.

[41] Guidetti P, Bussotti S (2002). Effects of seagrass canopy removal on fish in shallow Mediterranean seagrass (*Cymodocea nodosa* and *Zostera noltii*) meadows: a local-scale approach. Mar. Biol..

[42] Cuadros, A. *et al.* The three-dimensional structure of Cymodocea nodosa meadows shapes juvenile fish assemblages (Fornells Bay, Minorca Island). *Reg. Stud. Mar. Sci.* (2017).

[43] Francour P, Le Direac’h L (1994). Recrutement de l’ichtyofaune dans l’herbier superficiel à Posidonia oceanica de la réserve naturelle de Scandola (Corse, Méditerranée nord-occidentale): données préliminaires. Trav. Sci. Parc. Nat. Régional Corse.

[44] Francour, P. & Le Direac’h, L. Analyse spatiale du recrutement des poissons de l’herbier à Posidonia oceanica dans la réserve naturelle de Scandola (Corse, Méditerranée nord-occidentale). Contrat Parc Naturel Régional de la Corse & GIS Posidonie. *LEML Publ Nice* 1–23 (2001).

[45] Francour, P. & Le Direach, L. Le recrutement des poissons dans les herbiers à Posidonia oceanica : quels sont les facteurs influents ? in *XXXIX AFL Congress* 67–78 (1995).

[46] Le Direac’h L, Francour P (1998). Recrutement de Diplodus annularis (Sparidae) dans les herbiers de posidonie de la Réserve Naturelle de Scandola (Corse). Trav. Sci. Parc. Nat. Rég. Corse.

[47] Guidetti P (2000). Differences among fish assemblages associated with Nearshore Posidonia oceanica Seagrass Beds, Rocky–algal Reefs and unvegetated sand habitats in the Adriatic Sea. Estuar. Coast. Shelf Sci..

[48] Félix-Hackradt FC, Hackradt CW, Treviño-Otón J, Pérez-Ruzafa Á, García-Charton JA (2014). Habitat use and ontogenetic shifts of fish life stages at rocky reefs in South-western Mediterranean Sea. J. Sea Res..

[49] Félix-Hackradt FC (2013). Environmental determinants on fish post-larval distribution in coastal areas of south-western Mediterranean Sea. Estuar. Coast. Shelf Sci..

[50] Cheminée A (2013). Nursery value of Cystoseira forests for Mediterranean rocky reef fishes. J. Exp. Mar. Biol. Ecol..

[51] Cheminée A (2017). Juvenile fish assemblages in temperate rocky reefs are shaped by the presence of macro-algae canopy and its three-dimensional structure. Sci. Rep..

[52] Cuadros A (2019). Juvenile fish in Cystoseira forests: Influence of habitat complexity and depth on fish behaviour and assemblage composition. Mediterr. Mar. Sci..

[53] Hinz H, Reñones O, Gouraguine A, Johnson AF, Moranta J (2019). Fish nursery value of algae habitats in temperate coastal reefs. PeerJ.

[54] Thiriet PD (2016). Abundance and diversity of Crypto- and Necto-Benthic coastal fish are higher in marine forests than in structurally less complex macroalgal assemblages. PLoS ONE.

[55] Thiriet P (2014). Comparaison de la Structure des Peuplements de Poissons et des Processus Écologiques Sous-Jacents, Entre les Forêts de Cystoseires et des Habitats Structurellement Moins Complexes, dans l’Infralittoral Rocheux de Méditerranée Nord-Occidentale.

[56] Cheminée A (2017). Shallow rocky nursery habitat for fish: Spatial variability of juvenile fishes among this poorly protected essential habitat. Mar. Pollut. Bull..

[57] Mercader M (2018). Spatial distribution of juvenile fish along an artificialized seascape, insights from common coastal species in the Northwestern Mediterranean Sea. Mar. Environ. Res..

[58] Tournois J (2017). Lagoon nurseries make a major contribution to adult populations of a highly prized coastal fish. Limnol. Oceanogr..

[59] Cuadros A (2018). Settlement and post-settlement survival rates of the white seabream (*Diplodus sargus*) in the western Mediterranean Sea. PLoS ONE.

[60] Cheminée A, Francour P, Harmelin-Vivien M (2011). Assessment of Diplodus spp. (Sparidae) nursery grounds along the rocky shore of Marseilles (France, NW Mediterranean). Sci. Mar..

[61] Pastor J, Koeck B, Astruch P, Lenfant P (2013). Coastal man-made habitats: Potential nurseries for an exploited fish species, Diplodus sargus (Linnaeus, 1758). Fish. Res..

[62] Vigliola L (1998). Spatial and temporal patterns of settlement among sparid fishes of the genus Diplodus in the northwestern Mediterranean. Mar. Ecol.-Prog. Ser..

[63] Vigliola L, Harmelin-Vivien M (2001). Post-settlement ontogeny in three Mediterranean reef fish species of the Genus Diplodus. Bull. Mar. Sci..

[64] Cuadros A (2017). Seascape attributes, at different spatial scales, determine settlement and post-settlement of juvenile fish. Estuar. Coast. Shelf Sci..

[65] Morat F (2014). Diet of the Mediterranean european shag, Phalacrocorax aristotelis desmarestii, in a northwestern mediterranean area: a competitor for local fisheries?. Sci. Rep. Port. Cros. Natl. Park.

[66] Morat F (2014). Offshore–onshore linkages in the larval life history of sole in the Gulf of Lions (NW-Mediterranean). Estuar. Coast. Shelf Sci..

[67] La Mesa G, Louisy P, Vacchi M (2002). Assessment of microhabitat preferences in juvenile dusky grouper (*Epinephelus marginatus*) by visual sampling. Mar. Biol..

[68] Vacchi M, La Mesa G, Finoia MG, Guidetti P, Bussotti S (1999). Protection measures and juveniles of dusky grouper, Epinephelus marginatus (Lowe, 1834) (Pisces, Serranidae), in the Marine Reserve of Ustica Island (Italy, Mediterranean Sea). Mar. Life.

[69] Bodilis P, Ganteaume A, Francour P (2003). Presence of 1 year-old dusky groupers along the French Mediterranean coast. J. Fish Biol..

[70] Bodilis P, Ganteaume A, Francour P (2003). Recruitment of the dusky grouper (*Epinephelus marginatus*) in the north-western Mediterranean Sea. Cybium.

[71] Mercader M (2016). Observation of juvenile dusky groupers (*Epinephelus marginatus*) in artificial habitats of North-Western Mediterranean harbors. Mar. Biodivers..

[72] Raventos N, Macpherson E (2005). Environmental influences on temporal patterns of settlement in two littoral labrid fishes in the Mediterranean Sea. Estuar. Coast. Shelf Sci..

[73] Raventos N, Macpherson E (2005). Effect of pelagic larval growth and size-at-hatching on post-settlement survivorship in two temperate labrid fish of the genus Symphodus. Mar. Ecol. Prog. Ser..

[74] Macpherson E, Raventos N (2005). Settlement patterns and post-settlement survival in two Mediterranean littoral fishes: influences of early-life traits and environmental variables. Mar. Biol..

[75] Raventos N (2004). Effects of wave action on nesting activity in the littoral five-spotted wrasse, Symphodus roissali,(Labridae), in the northwestern Mediterranean Sea. Sci. Mar..

[76] Schunter C (2019). A novel integrative approach elucidates fine-scale dispersal patchiness in marine populations. Sci. Rep..

[77] Biagi F, Gambaccini S, Zazzetta M (1998). Settlement and recruitment in fishes: The role of coastal areas. Ital. J. Zool..

[78] Franco A (2006). Use of shallow water habitats by fish assemblages in a Mediterranean coastal lagoon. Estuar. Coast. Shelf Sci..

[79] Harmelin-Vivien ML (1985). Évaluation visuelle des peuplements et populations de Poissons: Méthodes et problèmes. Rev. Ecol. Terre Vie.

[80] Faillettaz R (2020). Spatio-temporal patterns of larval fish settlement in the northwestern Mediterranean Sea. Mar. Ecol. Prog. Ser..

[81] Le Direach, L. *et al. Programme NUhAGE : Nurseries, habitats, génie écologique, Rapport final. Contrat GIS Posidonie: MIO: P2A développement/Agence de l’Eau Rhône-Méditerranée-Corse-Conseil Général du Var*. 1–146 (2015).

[82] Orellana S, Hernández M, Sansón M (2019). Diversity of Cystoseira sensu lato (Fucales, Phaeophyceae) in the eastern Atlantic and Mediterranean based on morphological and DNA evidence, including *Carpodesmia* gen. emend. and *Treptacantha* gen. emend. Eur. J. Phycol..

[83] Ballesteros, E. Els vegetals i la zonació litoral: espècies, comunitats i factors que influeixen en la seva distribució. (1992).

[84] Medrano A (2020). Ecological traits, genetic diversity and regional distribution of the macroalga Treptacantha elegans along the Catalan coast (NW Mediterranean Sea). Sci. Rep..

[85] Clarke, K. R., Gorley, R. N., Somerfield, P. J. & Warwick, R. M. *Change in Marine Communities: An Approach to Statistical Analysis and Interpretation*. (Primer-E Ltd, 2001).

[86] Clarke, K. R. & Gorley, R. N. Primer v6: User Manual/Tutorial-Primer-E Ltd. (2006).

[87] Anderson, M., Gorley, R. & Clarke, K. *PERMANOVA+ for PRIMER: guide to software and statistical methods*. (Primer-e, 2008).

[88] R Core Team. *R: A language and environment for statistical computing*. (R Foundation for Statistical Computing, 2017).

[89] Wickham H (2009). ggplot2: Elegant Graphics for Data Analysis.

[90] August PV (1983). The role of habitat complexity and heterogeneity in structuring tropical mammal communities. Ecology.

[91] Wedding LM, Lepczyk CA, Pittman SJ, Friedlander AM, Jorgensen S (2011). Quantifying seascape structure: Extending terrestrial spatial pattern metrics to the marine realm. Mar. Ecol. Prog. Ser..

[92] Thiriet, P., Cheminée, A., Mangialajo, L. & Francour, P. How 3D complexity of macrophyte-formed habitats affect the processes structuring fish assemblages within coastal temperate seascapes? in *Underwater Seascapes* (eds. Musard, O. *et al*.) 185–199 (Springer, 2014).

[93] Cheminée A, Merigot B, Vanderklift MA, Francour P (2016). Does habitat complexity influence fish recruitment?. Mediterr. Mar. Sci..

[94] Mercader M (2019). Is artificial habitat diversity a key to restoring nurseries for juvenile coastal fish? Ex situ experiments on habitat selection and survival of juvenile seabreams. Restor. Ecol..

[95] Winemiller KO, Leslie MA (1992). Fish assemblages across a complex, tropical freshwater/marine ecotone. Environ. Biol. Fishes.

[96] Nagelkerken I (2000). Importance of mangroves, seagrass beds and the shallow coral reef as a nursery for important coral reef fishes, using a visual census technique. Estuar. Coast. Shelf Sci..

[97] Adams AJ (2006). Nursery function of tropical back-reef systems. Mar. Ecol. Prog. Ser..

[98] Vigliola L, Harmelin-Vivien M, Meekan MG (2000). Comparison of techniques of back-calculation of growth and settlement marks from the otoliths of three species of Diplodus from the Mediterranean Sea. Can. J. Fish. Aquat. Sci..

[99] Ventura D, Lasinio GJ, Ardizzone G (2015). Temporal partitioning of microhabitat use among four juvenile fish species of the genus Diplodus (Pisces: Perciformes, Sparidae). Mar. Ecol..

[100] Thibaut T, Blanfune A, Boudouresque CF, Verlaque M (2015). Decline and local extinction of Fucales in French Riviera: the harbinger of future extinctions?. Mediterr. Mar. Sci..

[101] Thibaut T, Pinedo S, Torras X, Ballesteros E (2005). Long-term decline of the populations of Fucales (*Cystoseira* spp. and *Sargassum* spp.) in the Alberes coast (France, North-western Mediterranean). Mar. Pollut. Bull..

[102] Thibaut T (2016). Unexpected temporal stability of cystoseira and sargassum forests in port-cros, one of the Oldest Mediterranean Marine National Parks. Cryptogam. Algol..

[103] Blanfuné A, Boudouresque CF, Verlaque M, Thibaut T (2016). The fate of Cystoseira crinita, a forest-forming Fucale (Phaeophyceae, Stramenopiles), in France (North Western Mediterranean Sea). Estuar. Coast. Shelf Sci..

[104] Sales M, Cebrian E, Tomas F, Ballesteros E (2011). Pollution impacts and recovery potential in three species of the genus Cystoseira (Fucales, Heterokontophyta). Estuar. Coast. Shelf Sci..

[105] Sala E, Boudouresque CF, Harmelin-Vivien M (1998). Fishing, trophic cascades, and the structure of algal assemblages: Evaluation of an old but untested paradigm. Oikos.

[106] Sala E, Kizilkaya Z, Yildirim D, Ballesteros E (2011). Alien marine fishes deplete algal biomass in the Eastern Mediterranean. PLoS ONE.

[107] Planes S (1999). Spatio-temporal variability in growth of juvenile sparid fishes from the Mediterranean littoral zone. J. Mar. Biol. Assoc. UK.

[108] Macpherson E (1997). Mortality of juvenile fishes of the genus Diplodus in protected and unprotected areas in the western Mediterranean Sea. Mar. Ecol. Prog. Ser..

[109] Pankhurst NW, Munday PL (2011). Effects of climate change on fish reproduction and early life history stages. Mar. Freshw. Res..

[110] Hidalgo M (2019). Accounting for ocean connectivity and hydroclimate in fish recruitment fluctuations within transboundary metapopulations. Ecol. Appl..

[111] Nagelkerken I, Sheaves M, Baker R, Connolly RM (2015). The seascape nursery: A novel spatial approach to identify and manage nurseries for coastal marine fauna. Fish Fish..

[112] Colloca F (2015). The seascape of demersal fish nursery areas in the North Mediterranean Sea, a first step towards the implementation of spatial planning for trawl fisheries. PLoS ONE.

[113] Cheminée, A., Feunteun, E., Clerici, S., Bertrand, C. & Francour, P. Management of infralittoral habitats: towards a seascape scale approach. in *Underwater Seascapes: From geographical to ecological perspectives* (eds. Musard, O., Francour, P. & Feunteun, E.) 240 (Springer, 2014).

[114] Grober-Dunsmore R, Pittman SJ, Caldow C, Kendall MS, Frazer TK (2009). A landscape ecology approach for the study of ecological connectivity across tropical marine seascapes. Ecol. Connect. Trop. Coast. Ecosyst..

[115] Meinesz A, Lefevre JR, Astier JM (1991). Impact of coastal development on the infralittoral zone along the southeastern Mediterranean shore of continental France. Mar. Pollut. Bull..

[116] Boudouresque, C. F. *et al.* The Management of Mediterranean Coastal Habitats: A Plea for a Socio-ecosystem-Based Approach. in *Evolution of Marine Coastal Ecosystems under the Pressure of Global Changes* (eds. Ceccaldi, H.-J. et al.) 297–320 (Springer, 2020).

[117] Seitz RD, Wennhage H, Bergström U, Lipcius RN, Ysebaert T (2014). Ecological value of coastal habitats for commercially and ecologically important species. ICES J. Mar. Sci..

[118] Boudouresque, C. F. *et al. Protection and conservation of Posidonia oceanica meadows. RAMOGE and RAC*. (SPA publisher, 2012).

[119] Sartoretto S (2017). An integrated method to evaluate and monitor the conservation state of coralligenous habitats: The INDEX-COR approach. Mar. Pollut. Bull..

[120] Meinesz A, Blanfuné A (2015). 1983–2013: Development of marine protected areas along the French Mediterranean coasts and perspectives for achievement of the Aichi target. Mar. Policy.

