# Supplementary material for: All shallow coastal habitats matter as nurseries for Mediterranean juvenile fish
Source: Sci Rep. 2021 Jul 16;11:14631. doi: 10.1038/s41598-021-93557-2 (PMC8285385; doi:10.1038/s41598-021-93557-2)
Supplement: Supplementary file 1 — Supplementary Information. [file 41598_2021_93557_MOESM1_ESM.docx]

**Title:**

All shallow coastal habitats matter as nurseries for Mediterranean juvenile fish

**Contributors :**

Adrien Cheminée^1,2*^, Laurence Le Direach^3^, Elodie Rouanet^3^, Patrick Astruch^3^, Adrien Goujard^3,5^, Aurélie Blanfuné^4^, Denis Bonhomme^3^, Laureline Chassaing^3^, Jean-Yves Jouvenel^6^, Sandrine Ruitton^4^, Thierry Thibaut^4^, Mireille Harmelin-Vivien^4^

**Supplementary tables**

Table S1. Mean values (±SE) of each of 26 habitat descriptors (see Table 2) of the shallow coastal fish nursery habitat types (number 1 to 7, see Table 1) in the NW Mediterranean Sea. Indications of significant seasonal variation (p<0.05) are shown in bold under the mean value. C: cold period; W: warm period.

|  | **RS (1)** |  | **AR**  **(2)** |  | **SB**  **(3)** |  | **CY**  **(4)** |  | **PO**  **(5)** |  | **POBR**  **(6)** |  | **POEX**  **(7)** |  |
| --- | --- | --- | --- | --- | --- | --- | --- | --- | --- | --- | --- | --- | --- | --- |
| **Depth** | 0.91 | (0.03) | 2.87 | (0.21) | 1.83 | (0.06) | 1.4 | (0.08) | 2.89 | (0.06) | 0.67 | (0.02) | 2.29 | (0.07) |
|  |  |  |  |  |  |  |  |  |  |  |  |  |  |  |
| **Slope** | 1.53 | (0.04) | 2.05 | (0.18) | 1.15 | (0.02) | 1.09 | (0.06) | 1.09 | (0.02) | 1.02 | (0.02) | 1.04 | (0.02) |
|  |  |  |  |  |  |  |  |  |  |  |  |  |  |  |
| **% Rocky substrate** | 84.28 | (1.48) | 0 |  | 0.35 | (1.11) | 0 |  | 44.98 | (3.06) | 0 |  | 13.11 | 2.54 |
|  |  |  |  |  |  |  |  |  |  |  |  |  |  |  |
| **% Blocks** | 12.92 | (1.37) | 100.00 | (0.00) | 1.59 | (0.37) | 0 |  | 0.40 | (0.38) | 0 |  | 0 |  |
|  |  |  |  |  |  |  |  |  |  |  |  |  |  |  |
| **% Pebbles** | 2.23 | (0.37) | 0 |  | 22.86 | (1.71) | 22.73 | (5.34) | 0 |  | 0 |  | 0 |  |
|  |  |  |  |  |  |  |  |  |  |  |  |  |  |  |
| **% Gravel** | 0.23 | (0.07) | 0 |  | **18.07** | **(1.40)** | 1.00 | (0.41) | 0 |  | 0.16 | (0.10) | 0 |  |
|  |  |  |  |  | **W<C** |  |  |  |  |  |  |  |  |  |
| **% Sand** | 0.30 | (0.10) | 0 |  | 55.34 | (2.10) | **76.27** | **(5.59)** | 54.62 | (3.07) | 99.60 | (0.18) | 86.80 | (3.17) |
|  |  |  |  |  |  |  | **W<C** |  |  |  |  |  |  |  |
| **% Mud** | 0.03 | (0.01) | 0 |  | 1.78 | (0.45) | 0 |  | 0 |  | 0.24 | (0.15) | 0.09 | (0.09) |
|  |  |  |  |  |  |  |  |  |  |  |  |  |  |  |
| **% *Posidonia*** | 0 |  | 0 |  | 0.01 | (0.01) | 0.22 | (0.22) | 95.09 | (0.58) | 63.18 | (2.56) | 92.76 | (0.86) |
|  |  |  |  |  |  |  |  |  |  |  |  |  |  |  |
| **% Dead Matte** | 0 |  | 0 |  | 0.26 | (0.17) | 6.78 | (1.89) | 2.47 | (0.37) | 33.44 | (2.42) | 4.69 | (0.64) |
|  |  |  |  |  |  |  |  |  |  |  |  |  |  |  |
| **% *Cymodocea*** | 0.11 | (0.07) | 0 |  | 0.15 | (0.06) | **80.44** | **(2.37)** | 0.08 | (0.04) | 1.44 | (0.35) | 0.01 | (0.01) |
|  |  |  |  |  |  |  | **W<C** |  |  |  |  |  |  |  |
| **% LowRugosity** | **37.93** | **(1.49)** | 51.50 | (7.16) | 0 | 0 | 0 |  | 2.20 | (0.84) | 0 |  | 0 |  |
|  | **W>C** |  |  |  |  |  |  |  |  |  |  |  |  |  |
| **% Medium Rugosity** | 22.57 | (1.16) | 11.50 | (3.35) | 1.99 | (0.65) | 0 |  | 0.34 | (0.20) | 0 |  | 0 |  |
|  |  |  |  |  |  |  |  |  |  |  |  |  |  |  |
| **% High Rugosity** | 26.68 | (1.28) | 1.00 | (0.69) | 0.35 | (0.26) | 0 |  | 0.11 | (0.08) | 0 |  | 0 |  |
|  |  |  |  |  |  |  |  |  |  |  |  |  |  |  |
| **%Very High Rugosity** | 11.11 | (0.76) | 11.00 | (2.80) | 0 |  | 0 |  | 0 |  | 0 |  | 0 |  |
|  |  |  |  |  |  |  |  |  |  |  |  |  |  |  |
| **% *Cystoseira* forest** | 21.41 | (1.37) | 0 |  | 0.30 | (0.15) | 0 |  | 0 |  | 0 |  | 0 |  |
|  |  |  |  |  |  |  |  |  |  |  |  |  |  |  |
| **% Arborescent algae** | 7.47 | (0.92) | 0 |  | 0.04 | (0.03) | 0 |  | 0.01 | (0.01) | 0 |  | 0 |  |
|  |  |  |  |  |  |  |  |  |  |  |  |  |  |  |
| **% Bushland** | 49.16 | (1.48) | 58.00 | (7.49) | 4.04 | (0.61) | 2.66 | (0.79) | 0.74 | (0.20) | 17.83 | (1.85) | 1.61 | (0.38) |
|  |  |  |  |  |  |  |  |  |  |  |  |  |  |  |
| **% Turf/Encrusting** | 16.82 | (1.00) | 42.00 | (7.49) | 5.52 | (0.81 | 1.18 | (0.42) | 0.39 | (0.10) | 8.39 | (1.53) | 2.08 | (0.42) |
|  |  |  |  |  |  |  |  |  |  |  |  |  |  |  |
| **% Wrecked algae** | 0.68 | (0.11) | 0 |  | 6.20 | (0.54) | 2.69 | (0.64) | 0.34 | (0.10) | 3.60 | (0.83) | 0.80 | (0.23) |
|  |  |  |  |  |  |  |  |  |  |  |  |  |  |  |
| **Height *Posidonia*** | na |  | na |  | na |  | na |  | **59.17** | **(1.30)** | **34.88** | **(1.78)** | **52.84** | **(1.34)** |
|  |  |  |  |  |  |  |  |  | **W>C** |  | **W>C** |  | **W>C** |  |
| **Height *Cymodocea*** | na |  | na |  | na |  | 9.16 | (0.18) | na |  | na |  | na |  |
|  |  |  |  |  |  |  |  |  |  |  |  |  |  |  |
| **Height *Cystoseira*** | **7.33** | **(0.18)** | na |  | 9.92 | (0.30) | na |  | na |  | na |  | na |  |
|  | **W>C** |  |  |  |  |  |  |  |  |  |  |  |  |  |
| **Height Arborescent** | 7.51 | (0.15) | na |  | na |  | na |  | na |  | na |  | na |  |
|  |  |  |  |  |  |  |  |  |  |  |  |  |  |  |
| **Height Bushland** | 5.33 | (0.10) | 5.39 | (0.85) | 5.76 | (0.11) | na |  | 6.38 | (0.20) | **6.52** | **(0.15)** | 4.17 | (0.07) |
|  |  |  |  |  |  |  |  |  |  |  | **W>C** |  |  |  |
| **Height Turf** | 3.72 | (0.09) | 3.83 | (0.10) | 3.83 | (0.13) | na |  | na |  | na |  | na |  |
|  |  |  |  |  |  |  |  |  |  |  |  |  |  |  |

Table S1 (end). Mean values (±SE) of each of 26 habitat descriptors (see Table 2) of the shallow coastal fish nursery habitat types (number 8 to 14, See Table 1) in the NW Mediterranean Sea. Indications of significant seasonal variation (p<0.05) are shown in bold under the mean value. C: cold period; W: warm period.

|  | **POIN (8)** |  | **POCY (9)** |  | **PODM (10)** |  | **IPR (11)** |  | **IPS**  **(12)** |  | **IPM (13)** |  | **IRS (14)** |  |
| --- | --- | --- | --- | --- | --- | --- | --- | --- | --- | --- | --- | --- | --- | --- |
| **Depth** | 1.29 | (0.05) | 1.35 | (0.02) | 1.12 | (0.06) | 2.28 | (0.11) | 2.90 | (0.08) | 0.76 | (0.03) | **4.32** | **(0.29)** |
|  |  |  |  |  |  |  |  |  |  |  |  |  | **W<C** |  |
| **Slope** | 1.00 | (0.00) | 1.00 | (0.00) | 1.00 | (0.00) | 1.48 | (0.06) | 1.11 | (0.03) | 1.00 | (0.00) | **2.05** | **(0.35)** |
|  |  |  |  |  |  |  |  |  |  |  |  |  | **W>C** |  |
| **% Rocky substrate** | 0 |  | 0 |  | 12.38 | (4.13) | 79.93 | (2.45) | 5.73 | (1.11) | **11.11** | **(4.74)** | 50.00 | (0.01) |
|  |  |  |  |  |  |  |  |  |  |  | **W>C** |  |  |  |
| **% Blocks** | 0 |  | 0 |  | 0.32 | (0.32) | 12.36 | (1.85) | 0.01 | (0.01) | 0 |  | 0 |  |
|  |  |  |  |  |  |  |  |  |  |  |  |  |  |  |
| **% Pebbles** | 0.79 | (0.27) | 0 |  | 0.16 | (0.16) | 4.23 | (0.76) | 0.03 | (0.03) | 0 |  | 0 |  |
|  |  |  |  |  |  |  |  |  |  |  |  |  |  |  |
| **% Gravel** | 2.67 | (1.00) | 0 |  | 0.08 | (0.08) | 0.47 | (0.21) | 0 |  | 0 |  | 0 |  |
|  |  |  |  |  |  |  |  |  |  |  |  |  |  |  |
| **% Sand** | 77.03 | (3.40) | 100 | (0.00) | **87.06** | **(4.22)** | 3.01 | (0.79) | 94.24 | (1.11) | 88.89 | (4.74) | 50.00 | (0.01) |
|  |  |  |  |  | **W<C** |  |  |  |  |  |  |  |  |  |
| **% Mud** | **19.51** | **(3.10).8** | 0 |  | 0 |  | 0 |  | 0 |  | 0 |  | 0 |  |
|  | **W<C** |  |  |  |  |  |  |  |  |  |  |  |  |  |
| **% *Posidonia*** | 0 |  | 0 |  | 0.71 | (0.42) | 48.94 | (0.41) | 47.46 | (0.29) | 50.11 | (0.25) | 0.05 | (0.05) |
|  |  |  |  |  |  |  |  |  |  |  |  |  |  |  |
| **% Dead Matte** | 0 |  | 0 |  | 94.89 | (0.97) | 1.75 | (0.41) | 3.98 | (0.57) | 49.89 | (0.25) | 0 |  |
|  |  |  |  |  |  |  |  |  |  |  |  |  |  |  |
| **% *Cymodocea*** | 1.66 | (0.43) | **87.61** | **(1.58)** | 0.35 | (0.32) | 0.49 | (0.21) | 1.96 | 0.35 | 0 |  | 0 |  |
|  |  |  | **W>C** |  |  |  |  |  |  |  |  |  |  |  |
| **% LowRugosity** | 0 |  | 0 |  | 0 |  | 31.20 | (2.71) | 1.05 | (0.74) | 0 |  | 42.14 | (9.16) |
|  |  |  |  |  |  |  |  |  |  |  |  |  | **W>C** |  |
| **% Medium Rugosity** | 0 |  | 0 |  | 0 |  | 24.27 | (1.99) | 0 |  | 0 |  | 20.48 | (5.45) |
|  |  |  |  |  |  |  |  |  |  |  |  |  |  |  |
| **% High Rugosity** | 0 |  | 0 |  | 0 |  | 24.09 | (1.57) | 0 |  | 0 |  | 8.10 | (3.13) |
|  |  |  |  |  |  |  |  |  |  |  |  |  |  |  |
| **%Very High Rugosity** | 0 |  | 0 |  | 0 |  | 11.68 | (1.16) | 0 |  | 0 |  | 5.48 | (2.72) |
|  |  |  |  |  |  |  |  |  |  |  |  |  |  |  |
| **% *Cystoseira* forest** | 0 |  | 0 |  | 0 |  | 4.60 | (0.78) | 0.01 | (0.01) | 0 |  | 0.19 | (0.06) |
|  |  |  |  |  |  |  |  |  |  |  |  |  |  |  |
| **% Arborescent algae** | 0 |  | 0.01 | (0.01) | 0 |  | 0.48 | (0.13) | 0.31 | (0.18) | 0.01 | (0.01) | 2.14 | (1.32) |
|  |  |  |  |  |  |  |  |  |  |  |  |  |  |  |
| **% Bushland** | 1.75 | (0.65) | 0.52 | (0.15) | 28.31 | (4.97) | 25.03 | (1.05) | 1.32 | (0.38) | 13.45 | (2.34) | 25.24 | (2.55) |
|  |  |  |  |  |  |  |  |  |  |  |  |  |  |  |
| **% Turf/Encrusting** | 0.02 | (0.01) | 0 |  | 27.73 | (4.75) | 14.76 | (1.03) | 0.69 | (0.17) | 17.19 | (3.08) | 13.45 | (2.38) |
|  |  |  |  |  |  |  |  |  |  |  |  |  |  |  |
| **% Wrecked algae** | 25.82 | (2.59) | 23.76 | (2.30) | 10.79 | (1.27) | 2.83 | (0.72) | 15.92 | (1.79) | 4.11 | (1.46) | 7.38 | (1.68) |
|  |  |  |  |  |  |  |  |  |  |  |  |  |  |  |
| **Height *Posidonia*** | na |  | na |  | na |  | **51.74** | **(1.61)** | **60.27** | **(1.80)** | **47.85** | **(2.13)** | na |  |
|  |  |  |  |  |  |  | **W>C** |  | **W>C** |  | **W>C** |  |  |  |
| **Height *Cymodocea*** | na |  | **8.67** | **(0.14)** | na |  | na |  | na |  | na |  | na |  |
|  |  |  | **W>C** |  |  |  |  |  |  |  |  |  |  |  |
| **Height *Cystoseira*** | na |  | na |  | na |  | 7.30 | (0.24) | na |  | na |  | **5.67** | **(0.13)** |
|  |  |  |  |  |  |  |  |  |  |  |  |  | **W>C** |  |
| **Height Arborescent** | na |  | na |  | na |  | na |  | na |  | na |  | 7.50 | (1.16) |
|  |  |  |  |  |  |  |  |  |  |  |  |  |  |  |
| **Height Bushland** | na |  | na |  | **5.33** | **(0.25)** | **5.66** | **(0.18)** | 5.33 | (0.33) | 4.55 | (0.31) | 5.40 | (0.50) |
|  |  |  |  |  | **W>C** |  | **W>C** |  |  |  |  |  |  |  |
| **Height Turf** | na |  | na |  | **1.97** | **(0.14)** | na |  | na |  | na |  | 2.67 | (0.11) |
|  |  |  |  |  | **W<C** |  |  |  |  |  |  |  |  |  |

Table S2. List of juvenile fish families recorded, names of taxa used in statistical analysis and list of species gathered in taxa groups. The common names of species or genus, according to Fishbase, were given in the last column.

| **Family** | **List of taxa** | **Species gathered in taxa** | **Common names** |
| --- | --- | --- | --- |
| Undetermined | Larvae |  |  |
| Bothidae | *Arnoglossus* sp. |  | Scaldfish |
| Atherinidae | *Atherina* sp. |  | Silverside |
| Blenniidae | Blenniidae | *Parablennius gattorugine* | Tompot blenny |
|  |  | *Parablennius pilicornis* | Ringnet blenny |
|  |  | *Parablennius rouxi* | Blenny |
| Sparidae | *Boops boops* |  | Bogue |
| Bothidae | *Bothus sp.* |  | Flounder |
| Callionymidae | *Callionymus* spp*.* | *Callionymus pusillus* | Sailfish dragonet |
|  |  | *Callionymus* sp. | Dragonet |
| Pomacentridae | *Chromis chromis* |  | damselfish |
| Labridae | *Coris julis* |  | Mediterranean rainbow wrasse |
| Sparidae | *Dentex dentex* |  | Common dentex |
| Moronidae | *Dicentrarchus labrax* |  | European seabass |
| Sparidae | *Diplodus annularis* |  | Annular seabream |
| Sparidae | *Diplodus puntazzo* |  | Sharpsnout seabream |
| Sparidae | *Diplodus sargus* |  | White seabream |
| Sparidae | *Diplodus vulgaris* |  | Common two-banded seabream |
| Serranidae | *Epinephelus marginatus* |  | Dusky grouper |
| Gobiidae | Gobiidae | *Gobius bucchichi* | Bucchich’s goby |
|  |  | *Gobius cobitis* | Giant goby |
|  |  | *Gobius geniporus* | Slender goby |
|  |  | *Gobius niger* | Black goby |
|  |  | Other Gobiidae | Goby |
| Labridae | *Labrus merula* |  | Brown wrasse |
| Labridae | *Labrus viridis* |  | Green wrasse |
| Gobiesocidae | *Lepadogaster* sp. |  | Clingfish |
| Sparidae | *Lithognathus mormyrus* |  | Sand steenbrass |
| Mugilidae | Mugilidae |  | Mullet |
| Mullidae | *Mullus* spp. | *Mullus barbatus* | Red mullet |
|  |  | *Mullus surmuletus* | Surmulet |
| Sparidae | *Oblada melanura* |  | Saddled seabream |
| Sparidae | *Pagellus* spp*.* | *Pagellus acarne* | Axillary seabream |
|  |  | *Pagellus bogaraveo* | Blackspot seabream |
|  |  | *Pagellus erythrinus* | Common pandora |
| Sparidae | *Sarpa salpa* |  | Salema |
| Sciaenidae | *Sciaena umbra* |  | Brown meagre |
| Scorpaenidae | *Scorpaena porcus* |  | Black scorpionfish |
| Serranidae | *Serranus cabrilla* |  | Comber |
| Serranidae | *Serranus scriba* |  | Painted comber |
| Soleidae | *Solea* sp. |  | Sole |
| Sphyraenidae | *Sphyraena viridensis* |  | Yellowmouth barracuda |
| Centracanthidae | *Spicara* sp. |  | Picarel |
| Sparidae | *Spondyliosoma cantharus* |  | Black seabream |
| Labridae | *Symphodus cinereus* |  | Grey wrasse |
| Labridae | *Symphodus* spp. | *Symphodus doderleini* | Wrasse |
|  |  | *Symphodus mediterraneus* | Axillary wrasse |
|  |  | *Symphodus melanocercus* | Wrasse |
|  |  | *Symphodus ocellatus* | Wrasse |
|  |  | *Symphodus roissali* | Five-spotted wrasse |
|  |  | *Symphodus rostratus* | Wrasse |
|  |  | *Symphodus tinca* | East Atlantic peacock wrasse |
| Syngnathidae | *Syngnathus* sp. |  | Pipefish |
| Labridae | *Thalassoma pavo* |  | Ornate wrasse |
| Trachinidae | Trachinidae |  | Weever |
| Triglidae | *Trigla* sp. |  | Gurnard |
| Tripterygiidae | Tripterygiidae |  | Triplefin |

Table S3. Mean (±SE) abundance of juvenile fishes (number of individuals/10m^2^) recorded in Mediterranean shallow coastal nurseries in warm and cold periods. Nb habitats: number of habitat types in which species were recorded. -: absent.

| **Taxa** | **Warm** | **Cold** | **Nb Habitats** |
| --- | --- | --- | --- |
| *Arnoglossus* sp. | 0.005 (0.003) | - | 1 |
| *Atherina* sp*.* | 4.374 (1.159) | 0.375 (0.166) | 12 |
| Blenniidae | 0.012 (0.004) | 0.003 (0.002) | 4 |
| *Boops boops* | 0.180 (0.180) | - | 1 |
| *Bothus* sp. | 0.004 (0.002) | - | 1 |
| *Callionymus* spp. | 0.002 (0.001) | 0.003 (0.002) | 2 |
| *Chromis chromis* | 0.050 (0.025) | 0.005 (0.004) | 3 |
| *Coris julis* | 0.260 (0.036) | 0.111 (0.028) | 8 |
| *Dentex dentex* | 0.007 (0.003) | - | 5 |
| *Dicentrarchus labrax* | *-* | 0.017 (0.017) | 1 |
| *Diplodus annularis* | 0.667 (0.196) | 0.015 (0.005) | 13 |
| *Diplodus puntazzo* | 0.153 (0.043) | 0.051 (0.014) | 9 |
| *Diplodus sargus* | 0.400 (0.058) | 0.113 (0.024) | 13 |
| *Diplodus vulgaris* | 0.195 (0.026) | 2.088 (0.404) | 14 |
| *Epinephelus marginatus* | 0.001 (0.001) | - | 1 |
| Gobiidae | 1.004 (0.284) | 3.294 (0.566) | 12 |
| *Labrus merula* | 0.006 (0.002) | 0.005 (0.003) | 5 |
| *Labrus viridis* | 0.006 (0.003) | - | 2 |
| *Larvae* | 3.413 (1.200) | 8.765 (4.719) | 13 |
| *Lepadogaster* sp. | - | 0.002 (0.002) | 1 |
| *Lithognathus mormyrus* | 0.031 (0.012) | - | 2 |
| Mugilidae | 0.278 (0.064) | 0.176 (0.057) | 5 |
| *Mullus* spp*.* | 0.073 (0.016) | - | 10 |
| *Oblada melanura* | 0.540 (0.097) | 0.224 (0.097) | 12 |
| *Pagellus* spp. | 1.016 (0.368) | 0.287 (0.114) | 7 |
| *Sarpa salpa* | 1.894 (0.351) | 2.813 (0.533) | 11 |
| *Sciaena umbra* | 0.001 (0.001) | - | 1 |
| *Scorpaena porcus* | - | 0.003 50.003) | 1 |
| *Serranus cabrilla* | 0.038 (0.006) | 0.020 (0.006) | 8 |
| *Serranus scriba* | 0.049 (0.007) | 0.005 (0.003) | 8 |
| *Solea* sp. | 0.001 (0.001) | - | 1 |
| *Sphyraena viridensis* | 0.016 (0.008) | - | 4 |
| *Spicara* sp. | 0.005 (0.004) | - | 2 |
| *Spondyliosoma cantharus* | 0.026 (0.013) | - | 5 |
| *Symphodus cinereus* | 0.024 (0.006) | 0.002 (0.002) | 6 |
| *Symphodus* spp. | 1.465 (0.200) | 0.322 (0.071) | 13 |
| *Syngnathus* sp. | 0.001 (0.001) | - | 1 |
| *Thalassoma pavo* | 0.006 (0.003) | 0.028 (0.013) | 2 |
| Trachinidae | 0.056 (0.034) | 0.002 (0.002) | 1 |
| *Trigla* sp. | - | 0.002 (0.002) | 1 |
| Tripterygiidae | 0.005 (0.002) | 0.003 (0.003) | 1 |
